# Direct toxicity of cigarette smoke extract on cardiac function mediated by mitochondrial dysfunction in Sprague-Dawley rat ventricular myocytes and human induced pluripotent stem cell-derived cardiomyocytes

**DOI:** 10.1371/journal.pone.0295737

**Published:** 2024-01-02

**Authors:** Sakiko Matsumura, Jumpei Yasuda, Takuya Notomi, Yoshihiro Suzuki, I-Shan Chen, Daichi Murakami, Muneki Hotomi, Tomoe Y. Nakamura

**Affiliations:** 1 Department of Pharmacology, Faculty of Medicine, Wakayama Medical University, Wakayama city, Wakayama, Japan; 2 R&D Headquarters Development Department, SIBATA Scientific Technology Ltd, Saitama, Japan; 3 Department of Otolaryngology Head and Neck Surgery, Faculty of Medicine, Wakayama Medical University, Wakayama city, Wakayama, Japan; University of Minnesota, UNITED STATES

## Abstract

Cigarette smoke has been recognized as a major risk factor for cardiovascular disease. However, its direct effects on rodent and human cardiomyocytes and its cellular mechanisms are not fully understood. In this study, we examined the direct effects of cigarette smoke extract (CSE) on contractile functions, intracellular Ca^2+^ dynamics, and mitochondrial function using cultured or freshly isolated rat ventricular myocytes and human induced pluripotent stem cell (iPS)-derived cardiomyocytes. In rat cardiomyocytes, CSE (≥0.1%) resulted in a time- and concentration-dependent cessation of spontaneous beating of cultured cardiomyocytes, eventually leading to cell death, which indicates direct toxicity. In addition, 1% CSE reduced contractile function of freshly isolated ventricular myocytes. Similar contractile dysfunction (declined spontaneous beating rate and contractility) was also observed in human iPS-derived cardiomyocytes. Regarding intracellular Ca^2+^ dynamics, 1% CSE increased the Ca^2+^ transient amplitude by greatly increasing systolic Ca^2+^ levels and slightly increasing diastolic Ca^2+^ levels. CSE also accelerated the decay of Ca^2+^ transients, and triggered spike-shaped Ca^2+^ transients in some cells. These results indicate that CSE causes abnormal Ca^2+^ dynamics in cardiomyocytes. Furthermore, CSE induced a cascade of mitochondrial dysfunctions, including increased mitochondrial reactive oxygen species, opening of mitochondrial permeability transition pore, reduction of mitochondrial membrane potential, and release of cytochrome c from mitochondria. These results suggest that CSE-induced contractile dysfunction and myocardial cell death is caused by abnormal Ca^2+^ dynamics and subsequent mitochondrial dysregulation, which would result in reduced bioenergetics and activation of cell death pathways.

## Introduction

Cigarette smoking is reported to be closely associated with chronic obstructive pulmonary disease [1] and systemic sclerosis [2]. Smoking has also been identified as a major risk factor for cardiovascular disease [2]. Among them, hypertension [3, 4], ischemic heart disease, and sudden cardiac death [5] are highly associated with smoking. Cohort studies have reported that cigarette smokers are more likely to develop ischemic heart disease than non-smokers [6]. Proposed mechanisms for the adverse cardiovascular effects of smoking in experimental animals and humans are based on vascular injury including endothelial cell damage [7], neutrophil activation and platelet aggregation [8], and atherosclerosis and atherosclerotic plaque disruption [9, 10] caused by these factors. Cigarette smoke contains thousands of various toxic components such as nicotine, tar, and reactive oxygen species, which have been proposed to cause vascular damage and indirectly lead to cardiovascular disease [10]. However, it is possible that cigarette smoke has a direct effect on cardiomyocytes. At the individual level, though, it is difficult to analyze whether these effects are direct or not, due to vascular responses and blood components. Therefore, there is a need to investigate the direct and acute effects of cigarette smoke extract (CSE) on isolated cardiomyocytes, but few studies have examined such effects or intracellular mechanisms in detail. Indicators of cardiac function include myocardial contractile and diastolic functions, and spontaneous beating rate. These are dependent on intracellular Ca^2+^ dynamics and are regulated by various ion channels and ion pumps, such as the sarcoplasmic reticulum (SR) Ca^2+^ release channel ryanodine receptor, the SR Ca^2+^ pump and its regulator phospholamban [11]. Myocyte function also depends on the function and integrity of mitochondria, which produce energy and regulate cell survival and death.

In the present study, we first examined the effects of CSE on contractile function, spontaneous beating rate, intracellular Ca^2+^ dynamics and various parameters of mitochondrial dysfunction in rat ventricular myocytes. However, considerable differences exist between rodent and human cardiomyocytes in terms of ion channel expression, beating rate, and energy metabolism. Therefore, we performed similar experiments using human induced pluripotent stem cell (iPS)-derived cardiomyocytes to determine whether the same phenomenon was observed in human cells.

## Material and methods

### Animals

This study conformed to the National Institutes of Health guidelines (Guide for the Care and Use of Laboratory Animals). All animal procedures were performed according to the Animal Welfare Committee guidelines of the Wakayama Medical University and were approved by the institutional ethics review board (approval reference number: 1000). Efforts were undertaken to minimize the number of animals and their suffering. Sprague Dawley rats used in this study were purchased from Japan SLC, Inc.

### Preparation of cigarette smoke extract (CSE) and determination of nicotine concentration

CSE was produced by bubbling a stream of smoke from 50 Hi-Lite cigarettes (Japan Tobacco Inc., Tokyo, Japan) into a 50-mL saline solution (1 cigarette/mL), which was defined as 100% CSE [12]. Such CSE was purchased from CMIC Pharmascience Corporation (Yamanashi, Japan) [13] and stored at –80 °C until use, as previously described [14]. Thus, 1% CSE is equivalent to 1/100 cigarettes/mL. We determined physiological/pathological range of CSE concentrations; nicotine concentration in 100% CSE was quantified using gas chromatography–mass spectrometry, a method based on ISO 18145:2003 [15]. In experiments using rat cardiomyocytes, modified Tyrode solution containing: NaCl, 137 mmol/L; KCl, 5.4 mmol/L; HEPES, 10 mmol/L; MgCl_2_, 1 mmol/L; CaCl_2_, 1.8 mmol/L and glucose, 10 mmol/L (adjusted to pH 7.4 with NaOH) was used as the control background solution. In experiments using human iPS-derived cardiomyocytes, phosphate buffered saline (PBS) was used as a control vehicle instead of CSE in the maintaining medium (Myoridge).

### Primary culture of neonatal rat ventricular myocytes (NRVMs)

Ventricles were excised from one-day-old neonatal rats. These were then dissociated into single myocytes by trypsinization and cultured in growth medium, as previously described [16, 17]. After excluding non-myocytes by differential adhesion treatment, myocytes were seeded into culture dishes and incubated in Dulbecco’s modified Eagle’s medium (DMEM) supplemented with 10% fetal bovine serum + 0.5% Penicillin/Streptomycin. Cardiomyocytes cultured for 3 days were used to measure spontaneous beating rate (equivalent to heart rate) because they beat spontaneously. They were also used for cytotoxicity evaluation by an [3-(4,5-dimethylthiazol-2-yl)-5-(3-carboxymethoxyphenyl)-2-(4-sulfophenyl)-2H-tetrazolium, inner salt] (MTS) assay, which requires a medium exchange step, because adherent cells are easier to handle.

### Isolation of adult rat ventricular myocytes (ARVMs)

Single ventricular myocytes were freshly isolated from adult male rat hearts through standard enzymatic techniques using Langendorff perfusion apparatus [18]. Briefly, hearts were rapidly removed from Isoflurane (3%)-anesthetized male Sprague Dawley rats (7–9-weeks old) and perfused in a Langendorff perfusion system with an enzyme solution containing 1 mg/mL type 1 collagenase (Sigma Aldrich, St. Louis, MO, U.S.A.) at 37 °C. Ventricles were cut into small pieces, and further digested by incubation with the enzyme solution described above in addition to 2 mg/ml BSA and 0.1 mmol/L Ca^2+^ for several minutes at 37 °C. The cell suspension was filtered (0.1 mm mesh), and maintained with gradually increasing Ca^2+^ (0.25, 0.5, 1 mmol/L) and finally resuspended in modified Tyrode solution. Myocytes were immediately plated onto laminin (0.1 mg/ml)-coated glass coverslips and allowed to attach by incubation at 37 °C. Freshly isolated cardiomyocytes were used to assess contractility and intracellular Ca^2+^ transients, as these parameters can be correctly measured using single cardiomyocytes rather than using cultured cell sheets.

### Evaluation of cytotoxicity

Cell viability was measured with an MTS assay using CellTiter 96 Aqueous One Solution Cell Proliferation Assay Kit (Promega Co., Wisconsin, U.S.A.) according to the manufacturer’s instructions as previously described [19]. Briefly, cultured NRVMs were incubated for 1 h with indicated concentrations (1–20%) of the CSE. After the incubation, 20 μl of the kit reagent was added to the cell culture in a 96-well plate and incubated for a further 2 h. The amount of the reduced form of MTS (formazan) was measured using a microplate reader by absorbance at 490 nm (SH9000Lab, CORONA ELECTRIC Co., Ltd., Ibaraki, Japan). MTS reduction activity of each concentration of CSE without cells was subtracted from those with cells and represented as percentages of the values obtained from 0% CSE-treated cells (control).

Additionally, two-color staining was also performed to assess cytotoxicity using a LIVE/DEAD^®^ viability/cytotoxicity assay kit (Thermo Fisher Scientific, Massachusetts, USA), following the manufacturer’s instructions. After 24 h of exposure to CSE, NRVMs were incubated with a staining solution containing calcein-AM and ethidium homodimer-1 for 30 min at RT (approximately 22 °C). After washing with PBS, the cells were observed using an All-in-One fluorescence microscope (BZ-X800; KEYENCE, Osaka, Japan). Calcein was retained inside living cells, emitting green fluorescence (ex/em: 495 nm/515 nm), while ethidium homodimer-1 passed through the injured cells and emitted red fluorescence in the nucleus of dead cells (ex/em: 495 nm/635 nm).

### Assessment of cardiac contractile function

To evaluate the spontaneous beating function of cardiomyocytes, we used an inverted microscope equipped with a 4K Hybrid Camera (Xcam4K Lite; Biotools, Gunma, Japan) and an incubation chamber (SV-140A; BLAST, Kanagawa, Japan) that maintained the temperature and CO_2_ concentration at 37 °C and 5%, respectively. To analyze the contractile and relaxation function of cardiomyocytes in more detail, an SI8000 Cell Motion Imaging System^®^ (SONY, Tokyo, Japan) was used. This system enables the quantitative analysis of the motion of beating cardiomyocytes. The maximum contraction and relaxation velocity are considered to correspond to the contractile and diastolic function of the cardiomyocytes. For cultured NRVMs, spontaneous beating rate (beats per minute) was measured to assess the beating function. Prior to measurement, cultured NRVM were incubated in serum-free DMEM + 0.5% penicillin/streptomycin for 2 h, followed by 1 h in Tyrode’s solution. For freshly isolated ARVMs, region of interests was set to surround the cardiomyocytes, after tracking the movement of cardiomyocytes contracting upon electrical stimulation, and the percentage of shortening relative to the long axis of the cell (fractional shortening) was calculated.

### Measurement of intracellular Ca^2+^ transient

Intracellular Ca^2+^ transient was measured as described previously [20]. Isolated ARVMs were loaded with a fluorescent Ca^2+^ indicator Fluo-8 acetoxymethyl ester (Invitrogen; 5 μmol/L for 40 min, 37°C). Cardiac contraction was elicited by electrical stimulation (1 Hz) under continuous perfusion of modified Tyrode’s solution at 37°C. Intracellular Ca^2+^ transients were measured by using the SI8000 Cell Motion Imaging System^®^ (SONY) with a 490-nm excitation wavelength and emission at 520 nm. Cytosolic [Ca^2+^] were expressed as the ratio of fluorescence intensity (F) at each time point relative to basal fluorescence (F_0_) before stimulation in each region (i.e., a value stable for at least 5 min in resting conditions).

In order to assess SR Ca^2+^ pump activity, the decay phase of each Ca^2+^ transient of cardiomyocytes before and after CSE treatment was fitted with a single exponential function and the time constant (τ) was obtained.

### Detection of mitochondrial reactive oxygen species (ROS) production

Cultured NRVMs were loaded with MitoSOX red (1 μM; Invitrogen), a mitochondria-targeted fluorescent indicator of superoxide production, and Hoechst 33342 (Invitrogen) for staining nuclei, in serum-free DMEM at 37 ◦C for 30 min. After washing, cells were exposed to 1% CSE in DMEM for 30 min. Fluorescence images (Ex/Em: 396/610 nm) were acquired and analyzed using an LSM900 confocal microscopy and ZEN3 imaging software (Carl Zwiss, Jena, Germany). Fluorescence intensity after 30 min of CSE exposure was normalized to the number of Hoechst-positive cells.

### Evaluation of change in mitochondrial membrane potential (ΔΨ_m_)

Isolated ventricular myocytes were loaded with 50 nM tetramethylrhodamine methyl ester (TMRM, Invitrogen) in Tyrode’s solution at 37 °C for 30 min. After washing, cells were exposed to 1% CSE for 20 min with electrical stimulation. Subsequently, fluorescence images (Ex/Em: 548/570 nm) were acquired 0, 10, and 20 min after CSE exposure and analyzed using the MetaMorph imaging software (Molecular Devices, San Jose, USA). The decrease in fluorescence intensity was used as an indicator of loss of mitochondrial membrane potential (ΔΨ_m_). Using the ratio of fluorescence intensity after 20 min of CSE exposure to that after 0 min of exposure, *box-and-whisker plots* and *scatter plots* were created using the SigmaPlot software (V. 14.5) (HULINKS, Tokyo, Japan).

### Evaluation of mitochondrial permeation transition pore (mPTP) opening

The mPTP assay was performed to evaluate mPTP opening using a commercially available kit (Cayman Chemical, Michigan, USA), which is based on the calcein/cobalt technique multiplexed with tetramethylrhodamine ethyl ester (TRME). Calcein/cobalt technique uses calcein AM to stain the entire cell, followed by a treatment with CoCl_2_ to quench the calcein fluorescence outside of the mitochondrial matrix. If the mitochondrial inner membrane (IMM) is intact, cobalt cannot penetrate the IMM and the mitochondrial matrix shows green fluorescence; if the IMM is compromised, calcein fluorescence is quenched and no fluorescence is observed. TMRE was used as an indicator of both membrane integrity and mitochondrial membrane potential. Briefly, NRVMs were loaded with 1 μM calcein-AM, 4 mM CoCl_2_, 20 nM TMRE and Hoechst 33342 (Invitrogen) in serum-free DMEM at 37°C for 30 min. After washing, cells were exposed to 1% CSE for 1 h in DMEM. Fluorescence images (Calcein Ex/Em: 485/535 nm and TMRE Ex/Em: 545/576 nm, respectively) were acquired and analyzed using LSM900 confocal microscopy and ZEN3 imaging software (Carl Zwiss). Calcein green fluorescence intensity in TMRE-positive regions (i.e., mitochondria) was measured 1 hour after CSE exposure.

### Evaluation of cytochrome c release from mitochondria by immunofluorescence analysis

Activation of the mitochondria-dependent cell death pathway was assessed by detection of cytochrome c release from mitochondria. This was done by immunofluorescence analysis using antibodies against cytochrome c and ATP synthase subunit C, which serves as a mitochondrial marker. Briefly, NRVMs were exposed to 1% CSE in serum-free DMEM for 1 h. After PBS washing, cells were fixed with 4% paraformaldehyde (10°C, 10 min) and permeabilized with 0.2% Triton-X 100 (room temperature, 1 min). After blocking with Blocking One Hist (Nacalai Tesque, Kyoto, Japan) for 10 min, NRVMs were treated with primary antibodies (anti-cytochrome c: mouse, 1/200, ab110325, and anti-ATP synthase subunit c: rabbit, 1/200, ab181243, abcam, Cambridge, UK) and incubated overnight at 4 °C. Secondary antibodies (Anti-mouse IgG Alexa fluor 488: 1/200, and Anti-rabbit IgG Alexa fluor 594, 1/200, Invitrogen) were then added, and nuclei were further stained with Hoechst 33342 (Invitrogen). Fluorescence images were acquired using LSM900 confocal microscopy and ZEN3 imaging software (Carl Zwiss). Colocalization of cytochrome C and ATP synthase subunit C in the myocytes treated with 1% CSE and Tyrode were analyzed by Pearson’s correlation using Image J.

### Preparation of human iPS-derived cardiomyocytes and functional analysis

Human iPS-derived cardiomyocytes (CarmyA: cardiac troponin T-positive highly differentiated cardiomyocytes [21]) were purchased (Myoridge, Kyoto, Japan). Cells that were at least 30 days after initiation of differentiation were used. The frozen cells were thawed and plated in seeding medium (Myoridge) in laminin-coated 24-well culture plates supplemented with 10 μM Y-27632, selective ROCK inhibotor (Nakalai Tesque, Kyoto, Japan). The culture medium was exchanged every day with a maintaining medium (Myoridge). A week after plating, cells were washed with PBS and detached using TrypLE (Thermo Fisher Scientific K.K., Tokyo, Japan). After centrifugation at 300 g for 5 min at room temperature (approximately 22 °C), cells were seeded in laminin-coated 96-well plates with seeding medium supplemented with 10 μM Y-27632. The medium was exchanged every day with a maintaining medium until the experiments for contractile function of the cells were conducted. To evaluate their contractile function, we used the SI8000 Cell Motion Imaging System^®^ as described above. The spontaneous beating rate and the contractile deformation distance (CDD), which is assumed to reflect the distance the myocardium moves during contraction and thus is an indicator of contractility of cardiomyocytes, were measured as previously described [22].

As human iPS-derived cardiomyocytes are very sensitive to medium replacement, especially with medium other than their maintaining medium (they stop beating for several hours when cultured in another solution such as Tyrode’s solution), maintaining medium was used as a background solution for contraction measurement. To evaluate the effect of CSE, human iPS-derived cardiomyocytes were cultured in new maintaining medium for 2 h, and 100% CSE or PBS was added directly to the medium to achieve the appropriate final concentration (0.1–5%).

### Statistical analysis

We used SigmaPlot software (V. 14.5), customed Image J (Fiji) and Free JSTAT (V. 22.0E). Differences between two groups were analyzed using paired and unpaired Student’s t-tests. A two-tailed P-value < 0.05 indicated statistical significance. Differences between more than two groups were analyzed using one-way analysis of variance followed by Dunnett’s test and two-way analysis of variance followed by Bonferroni’s test.

## Results

### CSE significantly decreased cell viability of cultured rat ventricular myocytes in a time- and concentration-dependent manner

**Fig 1:**
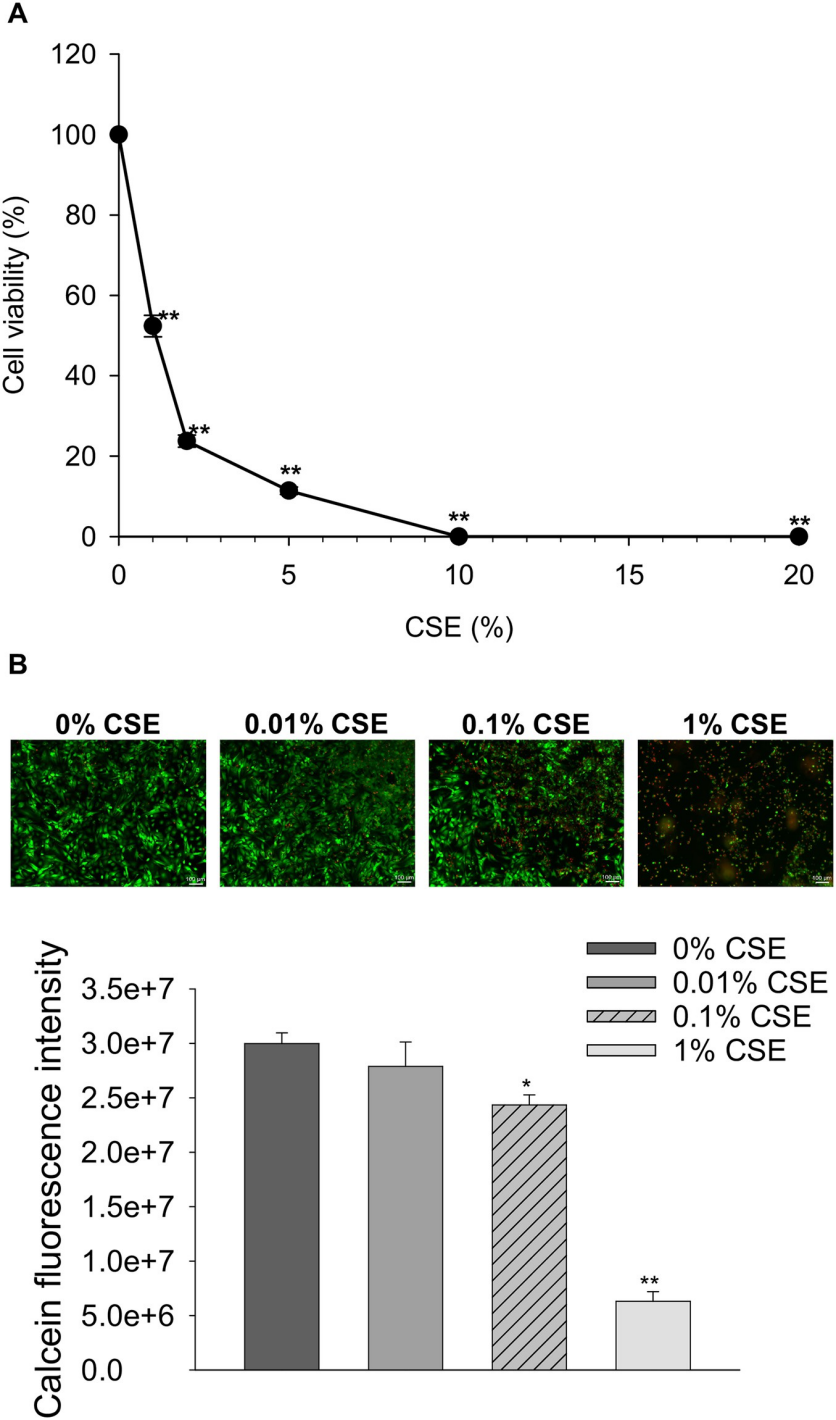
Effect of CSE on cell viability of cultured neonatal rat ventricular myocytes (NRVMs). (A) Cultured NRVMs were treated with 0, 1, 2, 5, 10, and 20% CSE for 1 h at 37 °C, and cell viability was assessed with an MTS assay. The normalized cell viabilities relative to the 0% CSE-treated control are shown as mean ± S.E.M. (n = 4 for each group). (B) Cultured NRVMs were treated with 0, 0.01, 0.1, and 1% CSE for 24 h at 37 °C, and cell viability was assessed with a LIVE/DEAD^®^ viability/cytotoxicity assay. The top panel shows representative fluorescent images of cardiomyocytes. Green fluorescence indicates viable cells that retained calcein, while red fluorescence indicates dead cell nuclei that were permeabilized by ethidium homodimer-1. The bottom panel is a quantitative analysis of calcein fluorescence intensity. Data are shown as mean ± S.E.M (n = 4 for each group). *P<0.05, **P<0.01 vs. 0% CSE-treated group.

We first examined the concentration-dependent cytotoxicity of CSE on cultured NRVMs. NRVMs were exposed to 0–20% CSE for 1 h at 37 °C, and cell viability was assessed by an MTS assay. Cell viability was significantly reduced compared to that in the vehicle control (0% CSE), indicating that CSE induces direct toxicity on cardiomyocytes (Fig 1A). To evaluate the effects of lower concentrations of CSE under longer exposure times, NRVMs were exposed to 0, 0.01%, 0.1%, and 1% CSE in a serum-free medium for 24 h. The LIVE/DEAD^®^ viability/cytotoxicity assay showed that exposure to 0.1% and 1% CSE significantly reduced the number of viable cells (Fig 1B). These data indicate that CSE decreases cell viability of ventricular myocytes in a time- and concentration-dependent manner.

### CSE decreased the spontaneous beating rate of cultured rat ventricular myocytes in a time- and concentration-dependent manner

**Fig 2:**
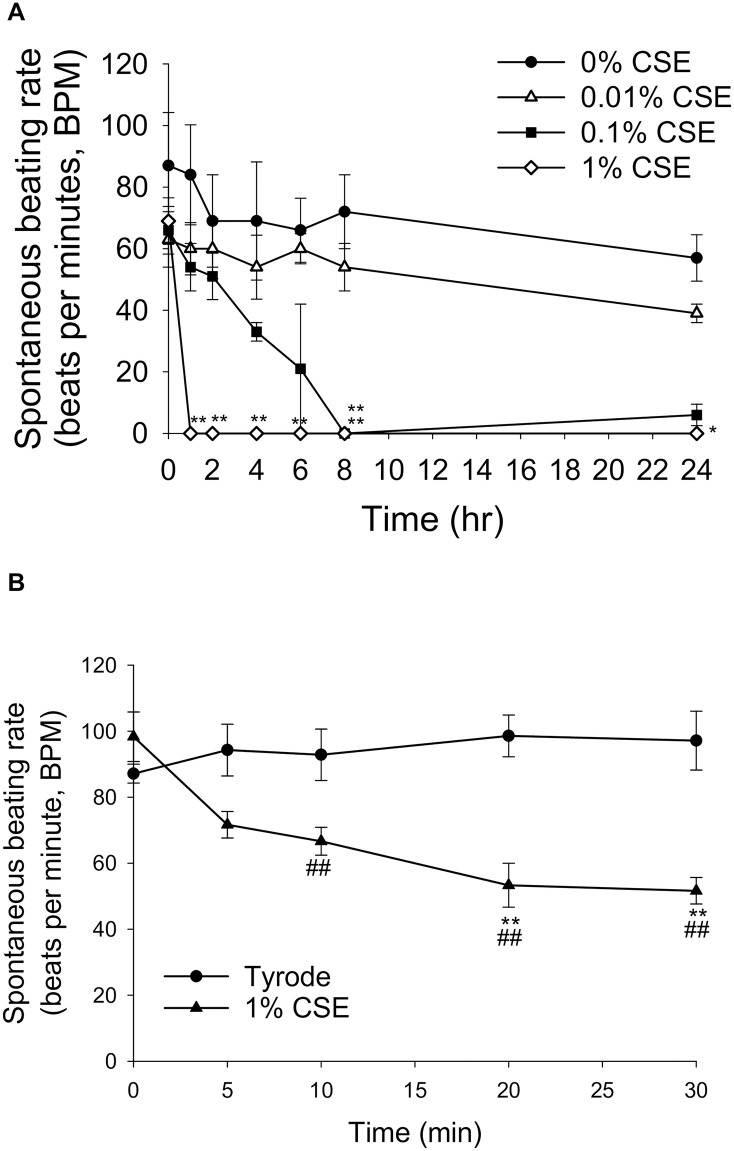
Effect of various concentrations of CSE on the spontaneous beating rate of cultured NRVMs. (A) Cultured NRVMs were treated with 0, 0.01, 0.1, and 1% CSE at 37 °C, and the spontaneous beating rate (BPM: beats per minute) was measured at 0, 1, 2, 4, 8 and 24 h. Data are shown as mean ± S.E.M. (n = 4 for each group). *P<0.05, **P<0.01 vs. 0% CSE. (B) NRVMs were treated with 0 and 1% CSE at 37 °C, and the spontaneous beating rate was measured every 5 min for 30 min. Data are shown as mean ± S.E.M (n = 4 for each group). **P<0.01 vs. Tyrode-treated group, ^##^*P*<0.01 vs. 0 min after exposure in the 1% CSE treated group.

Next, to determine the effect on cardiomyocyte function, we examined the effect of 0, 0.01, 0.1, and 1% CSE on the spontaneous beating rate of cultured NRVMs up to 24 h. As shown in Fig 2A, the spontaneous beating rate (BPM: beats per minute) of cardiomyocytes gradually decreased with 0.1% CSE, ceasing after 8 h exposure. When 1% CSE was used, the beating ceased within 1 h. To see more acute effects of CSE, NRVMs were treated with 0 and 1% CSE at 37 °C for 30 min. The spontaneous beating rate was gradually decreased (51.7 ± 3.66 BPM at 30 min vs. 98.3 ± 6.84 BPM at 0 min) and reached almost half of that in the time-matched control (Tyrode-treated) group after 30 min (51.7 ± 3.66 BPM with 1% CSE vs. 97.2 ± 8.26 BPM with Tyrode) (Fig 2B). These results indicate that CSE suppresses the spontaneous beating rate of NRVMs in a time- and concentration-dependent manner.

In order to know which concentration of CSE is physiologically relevant, the nicotine concentration in CSE was measured as an indicator. The results showed that 100% CSE contains 1.02 mg/mL nicotine. That is, 0.1% CSE contains about 1 ug/mL nicotine, a concentration that could be detected in the blood of heavy smokers, as reported in previous research [23]. Thus, 0.1% CSE is a physiologically appropriate concentration to use.

### CSE reduced the fractional shortening of freshly isolated rat ventricular myocytes

**Fig 3:**
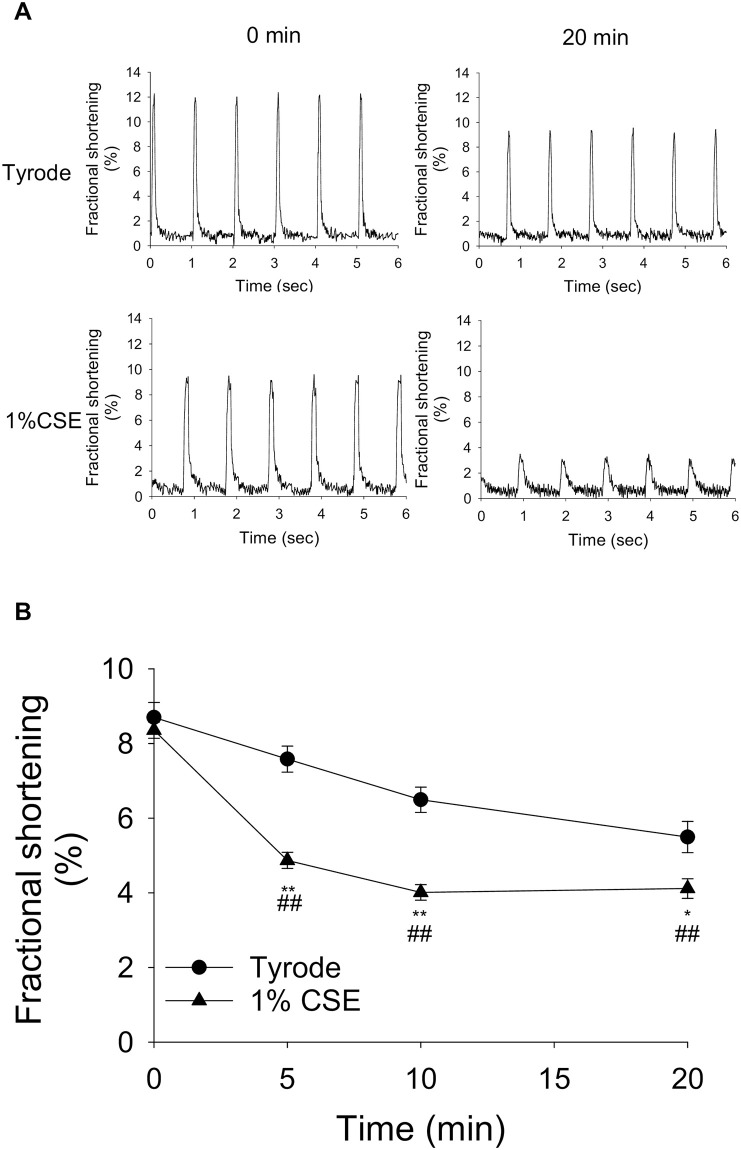
Effect of CSE on the contractile function of freshly isolated adult rat ventricular myocytes (ARVMs). The contraction of cardiomyocytes was elicited by electrical field stimulation (1 Hz) at 37 °C for 20 min. (A) Representative traces of cardiomyocytes before and 20 min after treatment with Tyrode or 1% CSE solution. (B) Time course of fractional shortening (%, percentage change in cardiomyocyte diameter during contraction) is shown as mean ± S.E.M. (n = 34 cells per group, from 4 experiments). **P<0.01 vs. Tyrode-treated group, ^##^*P*<0.01 vs. 0 min of 1% CSE treated group.

To examine the effect of CSE on the contractile function of cardiomyocytes, we next measured the fractional shortening of freshly isolated cardiomyocytes. The cardiomyocytes were electrically stimulated at 1 Hz and continuously perfused with Tyrode’s or CSE solution at 37 °C. Under these relatively harsh conditions, we observed that the cells were only energetic for < 1 h, even under the control Tyrode’s solution. Therefore, in subsequent experiments, we decided to use 1% CSE rather than 0.1% CSE as an exaggerated model to observe the effects of CSE and its intracellular mechanisms under favorable experimental conditions. As shown in the representative traces in Fig 3A, 1% CSE treatment for 20 min markedly decreased the fractional shortening of cardiomyocytes while the Tyrode treatment had smaller effects. From the analysis using multiple cells (n = 34), treatment with 1% CSE reduced fractional shortening of cardiomyocytes compared to the Tyrode-treated time-matched control, in a time-dependent manner, with significant effects at 5, 10 and 20 min after CSE exposure (Fig 3B). Because prolonged treatment with electrical stimulation is toxic to cardiomyocytes even under control conditions, the CSE effect was evaluated at times up to 20 min. There was also a significant reduction in the fractional shortening compared to 0 min (4.1 ± 0.26% at 20 min vs. 8.4 ± 0.21% at 0 min). These data suggest that CSE directly reduced the contractile function of ventricular myocytes.

### CSE altered intracellular Ca^2+^ dynamics in freshly isolated rat ventricular myocytes

**Fig 4:**
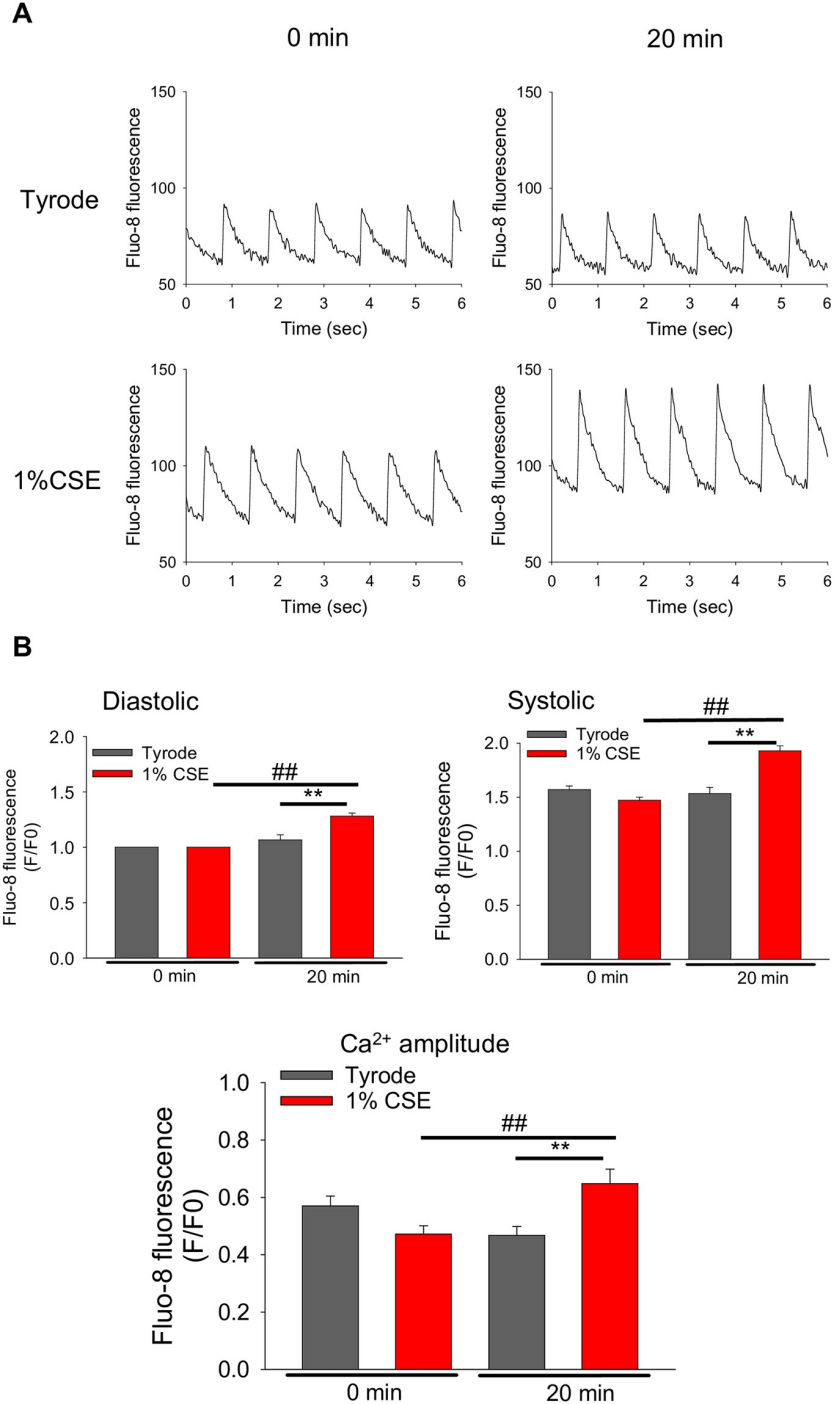
Effects of CSE on the intracellular Ca^2+^ dynamics in freshly isolated ARVMs. Intracellular Ca^2+^ transients of cardiomyocytes elicited by electrical field stimulation (1 Hz) at 37 °C before and 20 min after Tyrode or 1% CSE treatment. (A) Representative traces for each group. (B) Summary of diastolic and systolic Ca^2+^ levels, and Ca^2+^ transient amplitude obtained from each trace are shown as mean ± S.E.M. (Vehicle: n = 24 cells, 1% CSE: n = 21 cells, from 4 experiments). **P<0.01 vs. 20 min of Tyrode, ^##^*P*<0.01 vs. 0 min of 1% CSE-treated group.

Having determined that 1% CSE reduced the contractile function of cardiomyocytes, we next examined intracellular Ca^2+^ dynamics to explore the cellular mechanism. As shown in Fig 4A, there was little change in intracellular Ca^2+^ concentration 20 min after treatment of cardiomyocytes with Tyrode solution. In contrast, treatment of cardiomyocytes with 1% CSE for 20 min caused a large increase in systolic Ca^2+^ concentration and a small increase in diastolic Ca^2+^ concentration, resulting in a rise in Ca^2+^ transient amplitude (which is obtained by subtraction of the diastolic Ca^2+^ level from the systolic Ca^2+^ level) (Fig 4A). Analysis using multiple cells demonstrated that treatment with 1% CSE for 20 min significantly increased both systolic and diastolic Ca^2+^ levels and Ca^2+^-transient amplitudes (Fig 4B). Similar results were also obtained in another group of cardiomyocytes when exposed to 1% CSE for shorter periods of time (~6 min) (S1 Fig).

In addition, spike-shaped Ca^2+^ transients were often observed in the 1% CSE treatment. However, since this was also partially detected in the Tyrode group 20 min after the start of the experiment (data not shown), we compared the frequency of this type of Ca^2+^ transients after acute CSE treatments (~6 min), which had little effect in the Tyrode group (S2A Fig). Counting the incidence of spike-shaped Ca^2+^ transients, they occurred in 25.8% of the cardiomyocytes treated with 1% CSE for 6 min (4% in Tyrode). A significant increase in the mean number of spike-shaped Ca^2+^ transients (measured every 6 s) was also detected, although this was not detected at 0 min in both groups (S2B Fig). Taken together, these data indicate that CSE induces abnormal intracellular Ca^2+^ dynamics in ventricular myocytes.

**Fig 5:**
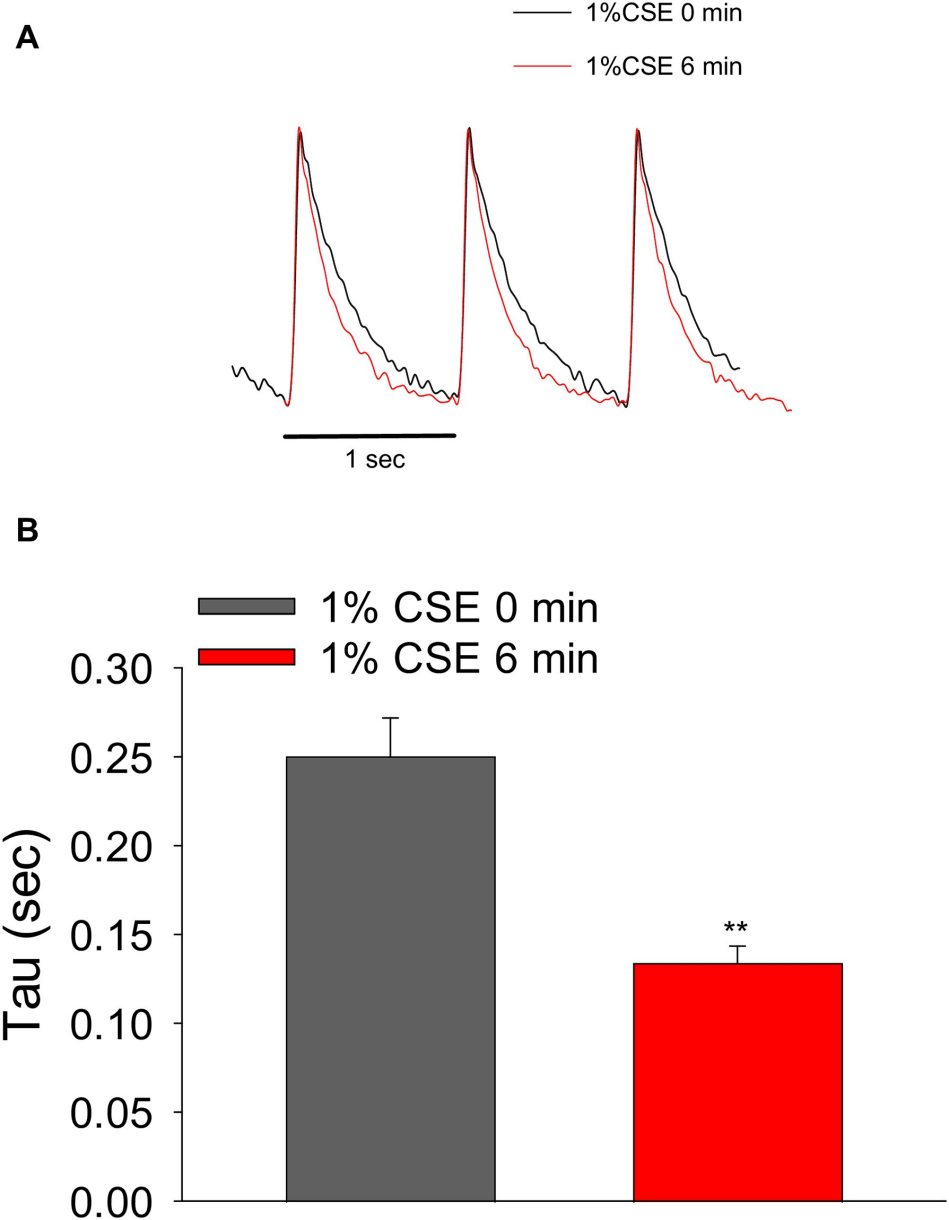
Kinetic analysis of the decay phase of Ca^2+^ transients in ARVMs treated with CSE. (A) Representative traces of normalized Ca^2+^ transients obtained from cardiomyocytes treated with 1% CSE for 0 and 6 min. Electrical stimulation-elicited Ca^2+^ transients were normalized to adjust for peak amplitude in cardiomyocytes before and after 1% CSE exposure. (B) The bar graph shows the time constant (τ) of the decay phase of the Ca^2+^ transient (n = 16 cells per group, from 4 experiments). **P<0.01 vs. 0 min of 1% CSE.

To further understand the molecular mechanisms underlying the above phenomenon, we performed a kinetic analysis of Ca^2+^ transients in cardiomyocytes before and after treatment with 1% CSE for 6 min. In rodent cardiomyocytes, the activity of Ca^2+^ uptake into the SR by the Ca^2+^ pump is largely reflected in the rate of decay of Ca^2+^ transients. Therefore, we investigated whether CSE treatment alters the rate of Ca^2+^ decay. Ca^2+^ transients that exhibited relatively normal shapes (i.e., no spike-shaped Ca^2+^ transients) were normalized to the peak amplitude before and after treatment with 1% CSE. As observed in Fig 5A, 1% CSE treatment for 6 min accelerated the decay of Ca^2+^ transients. Analysis using multiple cells showed that the time constant (τ) obtained by curve fitting of the decay phase of the Ca^2+^ transient was indeed reduced (0.1341 ± 0.0094 sec at 6 min vs. 0.2500 ± 0.0218 s at 0 min, n = 16 cells per group, from 4 experiments; Fig 5B). These results suggest that SR Ca^2+^ pump activity may have been enhanced by the 1% CSE treatment.

### CSE decreased the mitochondrial membrane potential (ΔΨ_m_) of freshly isolated rat ventricular myocytes

**Fig 6:**
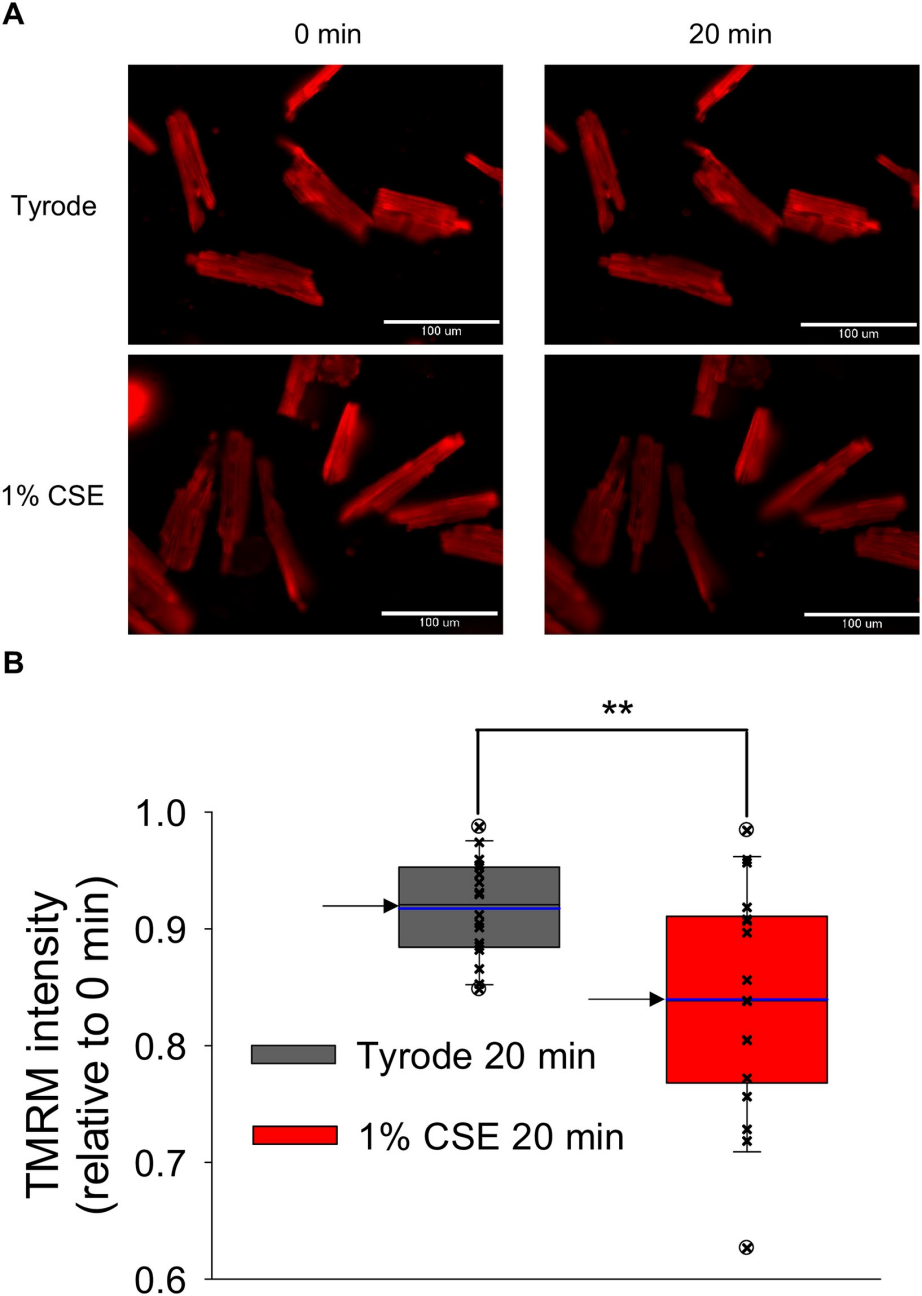
Effect of CSE on the mitochondrial membrane potential of freshly isolated ARVMs. Cardiomyocytes were loaded with 50 nM TMRM at 37 °C for 30 min and exposed to Tyrode or 1% CSE solution for 20 min with electrical stimulation. Fluorescence images were taken over time. (A) Representative images of cardiomyocytes before and 20 min after addition of CSE or Tyrode. (B) *Box-and-whisker plots* and *scatter plots* generated from the relative values of TMRM fluorescence intensity at 20 min to those at 0 min. The borders of *box plots* show the lower and upper quartile values (25% and 75%, respectively), while the lower and upper whiskers show the 10 and 90 percentiles, respectively. The blue line shows the mean value (n = 18 cells per group, from 4 experiments), and arrows show the median value of each group. White circles show outliers. **P<0.01 vs. Tyrode-treated group.

**Fig 7:**
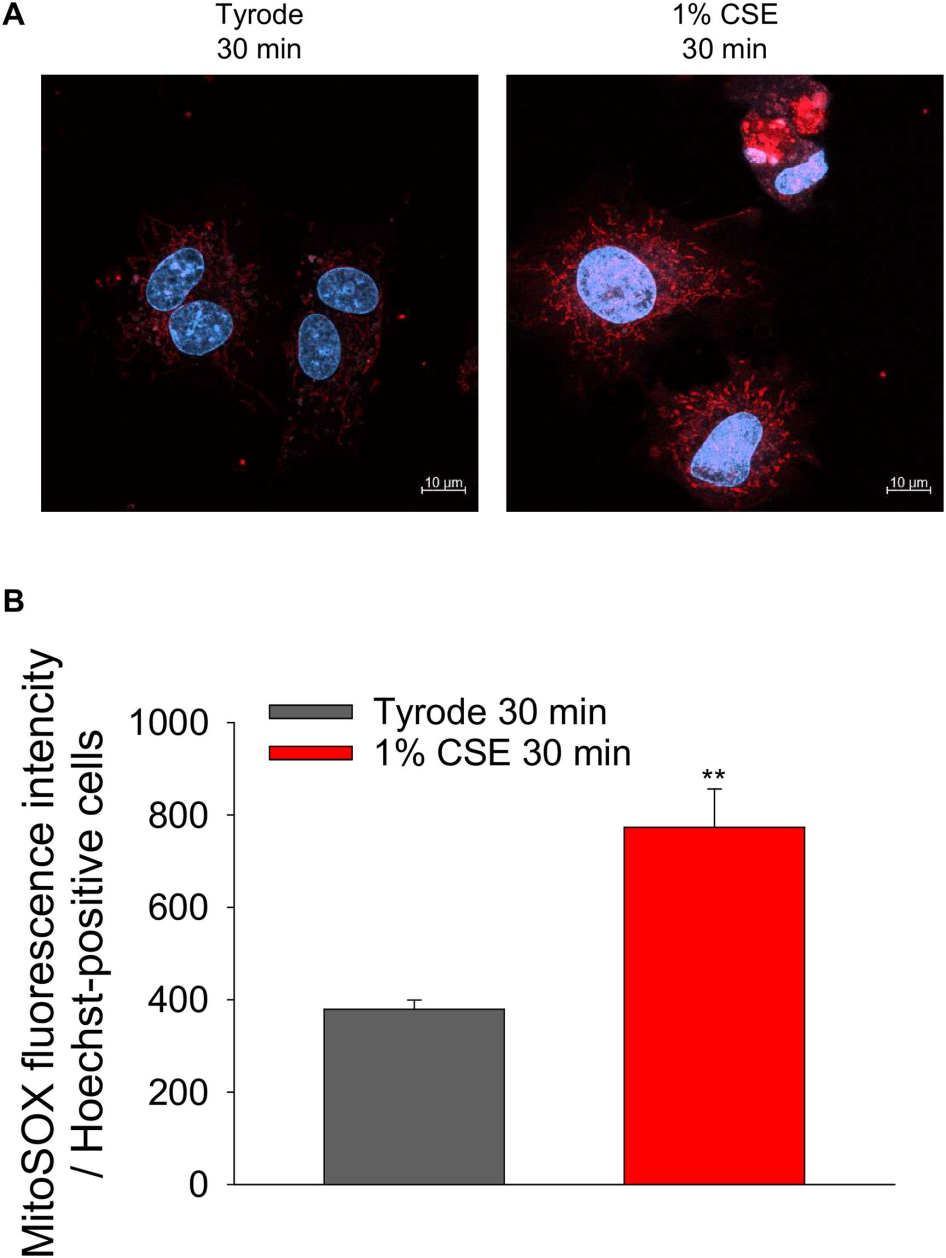
Effect of CSE on the mitochondrial ROS levels in cultured NRVMs. Cultured NRVMs were loaded with MitoSOX red, a mitochondria-targeted fluorescent indicator of superoxide production, and then treated with 1% CSE at 37 °C for 30 min. (A) Representative fluorescent images of cardiomyocytes. Red fluorescence indicates the mitochondrial ROS levels, and blue fluorescence indicates cell nuclei stained with Hoechst 33342. (B) Quantitative analysis of MitoSOX red fluorescence intensity normalized to Hoechst- positive cells. Data are shown as mean ± S.E.M (Vehicle: n = 40, 1% CSE: n = 24, from 4 experiments). **: *P*<0.01 vs. Tyrode-treated group.

To determine whether CSE impairs mitochondrial function in cardiomyocytes, we evaluated ΔΨ_m_ as its dissipation is an important early event in cellular damage. As shown in Fig 6, ΔΨ_m_ was monitored by TMRM fluorescence in the cardiomyocytes treated with Tyrode or 1% CSE for 20 min. Representative images show that the TMRM fluorescence was slightly decreased in CSE-treated cardiomyocytes compared to that in the Tyrode-treated group. *Box-and-whisker plots* were generated from the relative values of TMRM fluorescence intensity at 20 min to those at 0 min, with median values of 0.92 and 0.82 and average values of 0.92±0.01 and 0.84±0.02 for Tyrode and CSE groups, respectively (*P*<0.01) (Fig 7B). These results indicate that ΔΨ_m_ loss occurred in the CSE-treated group.

### CSE leads to mitochondrial dysfunction and mitochondrial cell death signaling pathways in cultured ventricular cardiomyocyte

The CSE-induced decrease in ΔΨm (Fig 6) suggest that the effects of CSE are due to mitochondrial dysfunction. This would reduce energy production and activate cell death pathways, which may be the mechanism for the observed cardiomyocyte contractile dysfunction. To determine whether this pathway is also activated in cultured NRVMs, we first measured mitochondrial reactive oxygen species (ROS) production, which is an early event induced by abnormal Ca^2+^ dynamics and induces mitochondrial dysfunction. Mitochondrial ROS were detected by MitoSOX red fluorescence in NRVMs treated with Tyrode or 1% CSE for 30 minutes, conditions that cause contractile dysfunction in about half of the cells (Fig 2). As observed in Fig 7, MitoSOX red fluorescence was significantly increased in CSE-treated cardiomyocytes compared to Tyrode -treated group, indicating that CSE treatment increased mitochondrial ROS.

**Fig 8:**
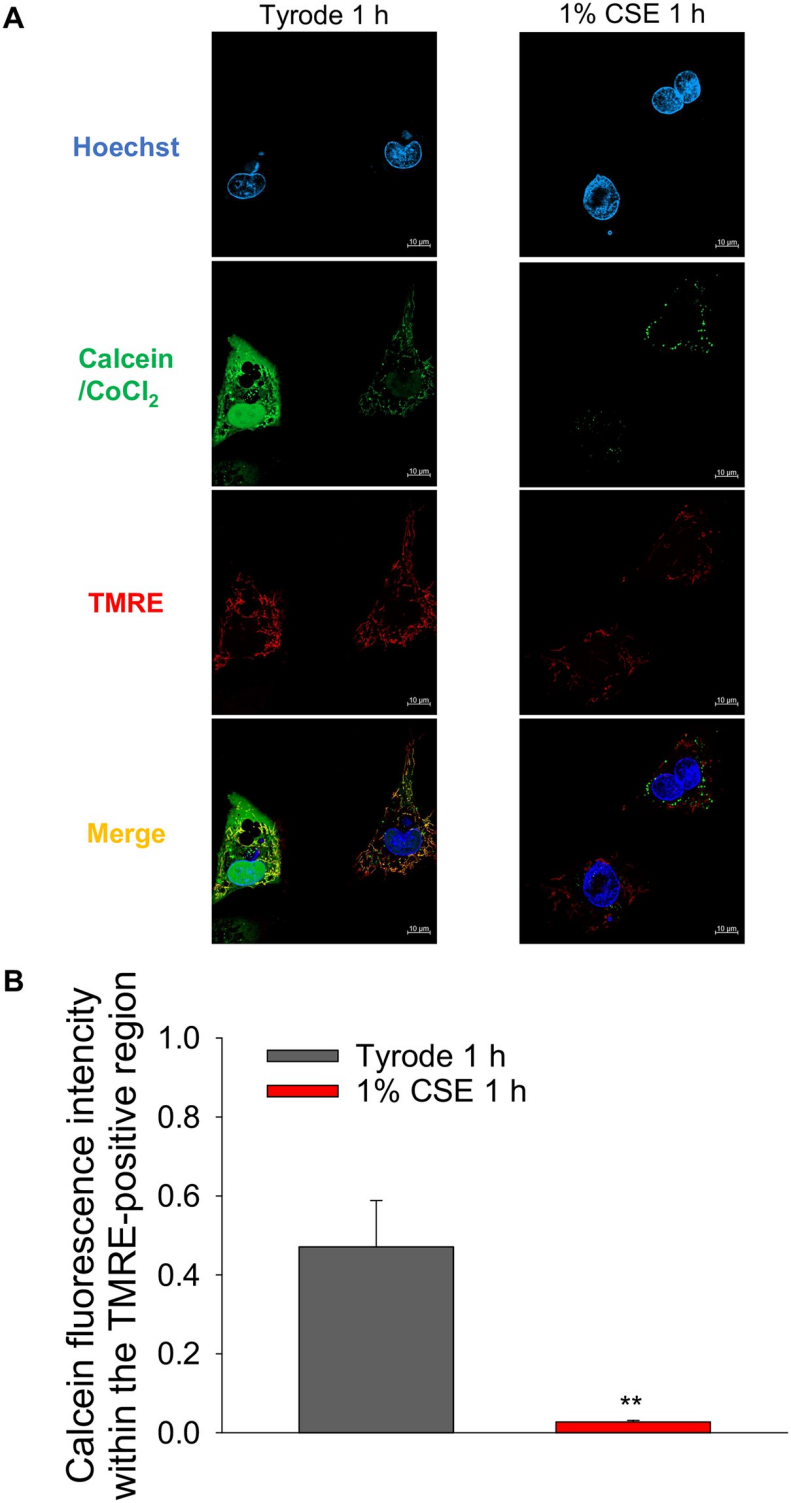
Effect of CSE on the opening of mitochondrial permeability transition pore (mPTP) of cultured NRVMs. Cultured NRVMs were loaded with calcein-AM, CoCl_2_ to quench calcein outside of the mitochondria, TMRE, and Hoechst 33342. Cells were then treated with 1% CSE at 37 °C for 1 h and opening of mPTP was assessed. (A) Representative fluorescent images of cardiomyocytes. Green fluorescence indicates calcein in mitochondria, TMRE red fluorescence indicates mitochondrial location and membrane potential, and blue fluorescence indicates cell nuclei. (B) Quantitative analysis of calcein fluorescence intensity in TMRE-positive regions, i.e., mitochondria. Data are shown as mean ± S.E.M (Vehicle: n = 21 cells, 1% CSE: n = 24 cells, from 4 experiments). **: *P*<0.01 vs. Tyrode-treated group.

Increased Ca^2+^ and ROS both activate a large conductance channel in the mitochondrial inner membrane, the mitochondrial permeability transition pore (mPTP), whose opening disrupts the mitochondrial membrane potential (ΔΨ_m_) [24]. We thus examined whether CSE treatment induces mPTP opening by assessing the attenuation of calcein fluorescence from mitochondrial regions labeled by TMRE (see methods). Representative images show that calcein green fluorescence and TMRE red fluorescence co-localized in Tyrode-treated NRVMs (Fig 8A, left column). On the other hand, calcein green fluorescence was reduced in the TMRE red positive region in CSE-treated NRNMs (Fig 8A, right column), indicating that CSE treatment released calcein from mitochondria. Note that TMRE red fluorescence intensity was decreased in CSE treated cultured cardiomyocytes, as observed in freshly isolated cardiomyocytes (Fig 7). Multiple-cell analysis demonstrated that treatment with 1% CSE for 1 h significantly reduced the calcein green fluorescence in mitochondria (Fig 8B), suggested that CSE induces opening of mPTP.

**Fig 9:**
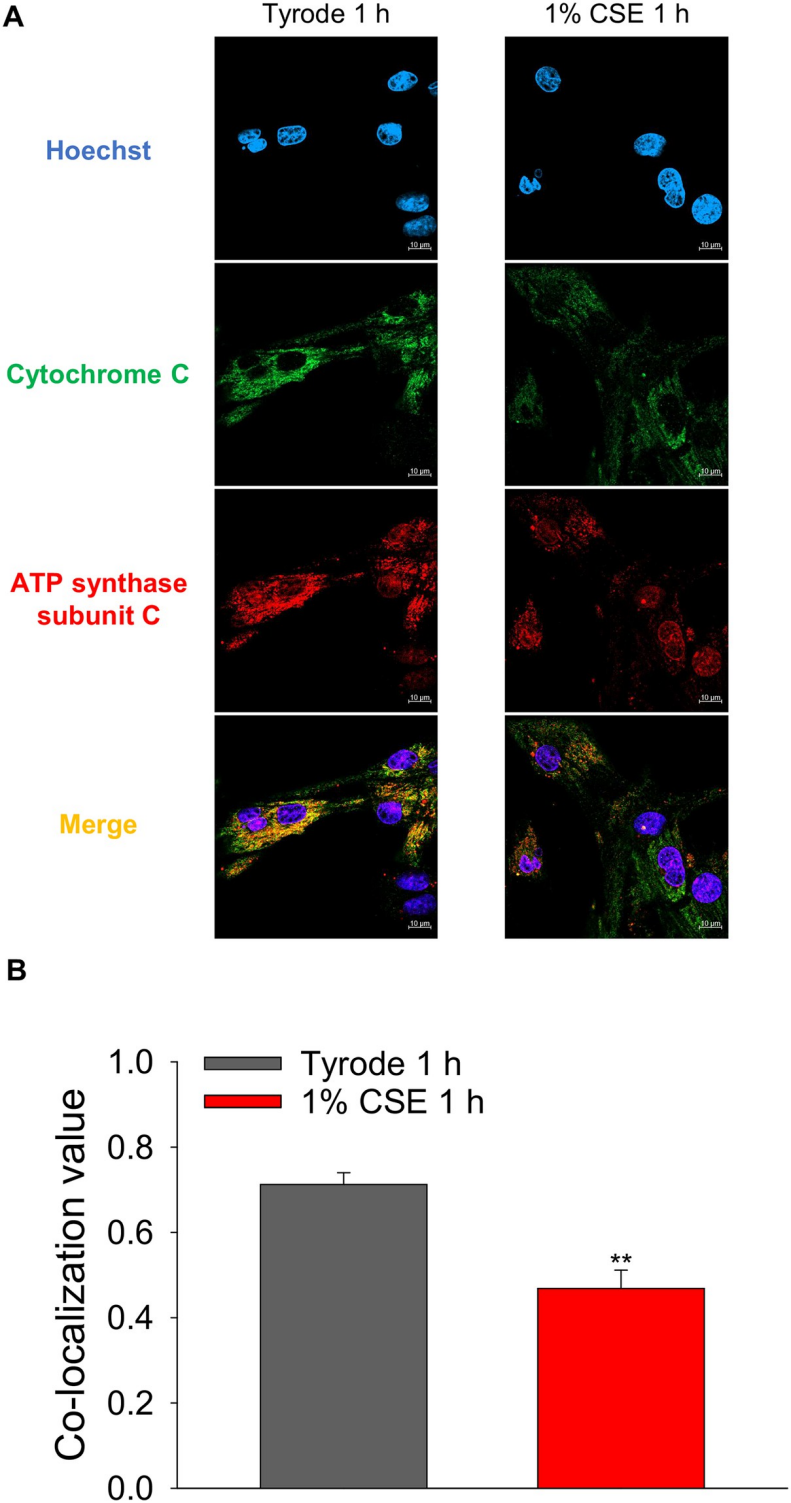
Effect of CSE on cytochrome c release from mitochondria of cultured NRVMs. A: Cultured NRVMs were treated with 1% CSE at 37 °C for 1 h, and immunofluorescence staining was preformed to detect co-localization of cytochrome c with ATP synthase subunit C. (A) Representative fluorescent images of cardiomyocytes. Green fluorescence indicates cytochrome c localization, red fluorescence indicates mitochondria- specific ATP synthase subunit C, and blue fluorescence indicates cell nuclei. (B) Quantitative analysis of co-localization values of cytochrome c and ATP synthase subunit C by Pearson’s correlation. Data are shown as mean ± S.E.M (Vehicle: n = 11, 1% CSE: n = 12, from 4 experiments). **: *P*<0.01 vs. Tyrode-treated group.

The opening of mPTP causes cytochrome c release from mitochondria, leading to activation of the mitochondria-dependent cell death pathway [25]. We therefore examined whether CSE induces cytochrome c release from mitochondria by immunofluorescence analysis using antibodies against cytochrome c and the mitochondrial marker ATP synthase subunit C. Fig 9A shows that in NRVMs treated with Tyrode for 1 h cytochrome c is mostly co-localized with ATP synthase subunit C, i.e., cytochrome c is within the mitochondria. On the other hand, in NRVMs treated with 1% CSE for 1 h, the localization of cytochrome c dissociated from ATP synthase subunit C. Co-localization analysis using the Pearson correlation method with multiple cells showed that CSE treatment significantly reduced the co-localization values of cytochrome c and ATP synthase subunit C (Fig 9B). This indicates that CSE induces cytochrome c release from mitochondria and may activate subsequent mitochondria-dependent cell death signaling.

### CSE significantly decreased the spontaneous beating rate and the contractility of human iPS-derived cardiomyocytes

**Fig 10:**
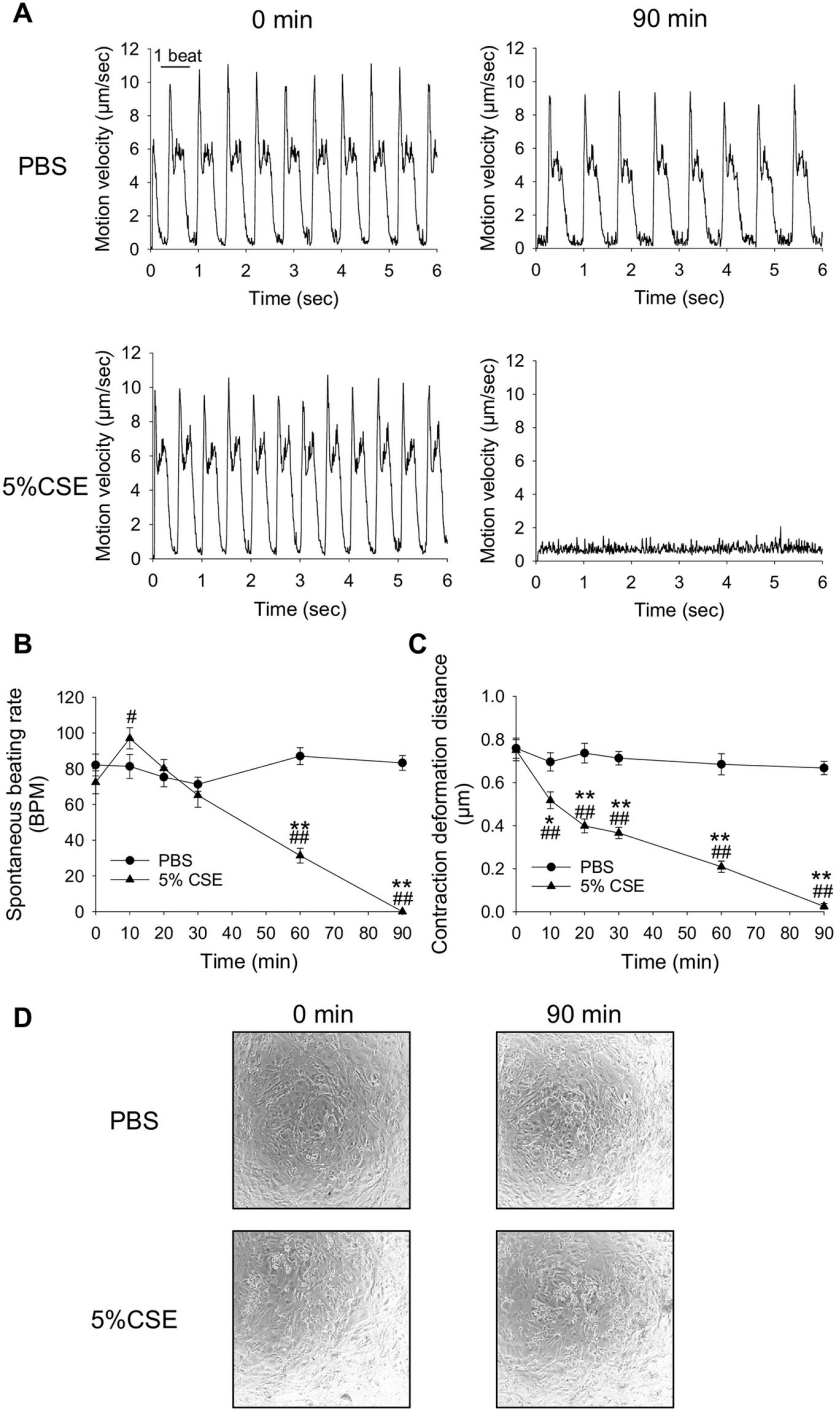
Effect of CSE on the spontaneous beating rate and contractility of human iPS-derived cardiomyocytes. Human iPS-derived cardiomyocytes incubated in cultured maintaining medium were exposed to 5% CSE or corresponding amounts of PBS (37 °C), and contractile functions were measured at 0, 10, 20, 30, 60, and 90 min. (A) Representative traces at 0 and 90 min for each group. As observed in the 90 min PBS treatment group, there were eight beats in 6 s. Each beat had two peaks, the first representing the contraction velocity and the second the relaxation velocity. However, in the 90 min 5% CSE treatment group, there were no beats. (B) The time course of spontaneous beating rate (BPM) for each group. (C) The contraction deformation distance (CDD) is the area calculated from the contraction velocity and time, and is an indicator of the contraction force. Data are shown as mean ± S.E.M. (PBS: n = 14, 5% CSE: n = 14). *P<0.05 vs. PBS-treated group, ^#^*P*<0.05 vs. 0 min of 5% CSE treated group. (D) The morphologies of human iPS-derived cardiomyocytes before and after 90 min exposure to PBS or 5% CSE.

To evaluate whether similar contractile dysfunctions are observed in human cardiomyocytes, we used human iPS-derived cardiomyocytes. When human iPS-derived cardiomyocytes in maintaining medium were treated with 1% CSE for 90 min, minimal changes were observed in the spontaneous beating rate or contraction deformation distance (CDD), which is an indicator of cardiomyocyte contractility (S3A and S3B Fig). However, when they were treated with 5% CSE, their spontaneous beating rate gradually decreased after a transient increase, and finally reached zero after 90 min (Fig 10A and 10B). In addition, 5% CSE treatment decreased the CDD in a time-dependent manner (Fig 10C).

Furthermore, the long-term (up to 2 days) effects of low doses of CSE were also examined. As mentioned above, treatment with 1% CSE for 90 min had little effect on contractile function of human iPS-derived cardiomyocytes. However, CDD was significantly decreased after prolonged treatment such as 360 min (S3B Fig), and spontaneous beating was arrested after treatment for more than 24 h (S3C and S3D Fig). 0.1% CSE had no effect after 48 hours of administration (S4A–S4D Fig), but longer administration might cause contractile dysfunction. These data indicate that CSE decreases contractile function of human iPS-derived cardiomyocytes in a time- and concentration-dependent manner.

Although contractile function was significantly reduced by 90 min treatment with 5% CSE (Fig 10A–10C), myocyte morphology was unchanged (Fig 10D), suggesting that CSE-induced contractile dysfunction was not due to cell death at this time point.

## Discussion

In the present study, we evaluated the direct effects of CSE on cell viability, contractile function, intracellular Ca^2+^ dynamics, and mitochondrial function in rat cardiomyocytes and human iPS-derived cardiomyocytes for contractile function. *In vivo*, the only cells directly exposed to cigarette smoke are the alveolar epithelium, while other cells, such as cardiomyocytes, are exposed to substances in cigarette smoke dissolved in the circulating blood. As this is the same form as CSE, a water-soluble extract of cigarette smoke, we believe that CSE is a suitable model system that closely mimics the reaction that occurs *in vivo*. We found that CSE decreased cardiomyocyte viability in a time and concentration-dependent manner (Fig 1), indicating that CSE has direct toxic effects. In addition, CSE at concentrations of 0.1% or higher markedly reduced the spontaneous beating rate of cultured NRVMs, eventually causing them to stop beating. (Fig 2). Our quantitative analysis indicated that 0.1% CSE contains approximately 1 μg/mL nicotine. The nicotine levels in the plasma of a person after smoking a cigarette can vary widely depending on several factors, including the type of cigarette, frequency of smoking, and individual differences in metabolism. However, the average concentration of nicotine after smoking one cigarette for 5 min has been reported to be near 50 ng/ml in arterial vessels [23]; thus, 0.1% CSE is equivalent to that of approximately 20 cigarettes, a detectable amount in heavy smokers, who smoke greater than or equal to 25 or more cigarettes a day [26]. Thus, we consider that it is physiologically appropriate to look at the effects of 0.1% CSE. However, as previously mentioned, to identify the effects and mechanisms of CSE in healthy cardiomyocytes, we used 1% CSE as an exaggerated model based on the condition that its effects are concentration dependent (Figs 1 and 2).

In freshly isolated cardiomyocytes, a significant decrease in contractile function was observed upon 5–20 min treatment of 1% CSE in electrically stimulated cardiomyocytes (Fig 3). Because knowledge of intracellular Ca^2+^ dynamics is critical for studying the mechanism of contraction, detailed analyses of the effect of CSE on Ca^2+^ transients were performed. We observed that 1% CSE treatment increased the amplitude of Ca^2+^ transients with large rises in systolic Ca^2+^ levels and small (or little) increases in diastolic Ca^2+^ levels (Fig 4 and S1 Fig).

In cardiac myocytes, electrical stimulation activates voltage-gated Ca^2+^ channels, causing a small amount of Ca^2+^ to enter the myocyte. This triggers the release of a large amount of Ca^2+^ from the SR via ryanodine receptor Ca^2+^ release channels, resulting in muscle contraction. Cytoplasmic Ca^2+^ is then taken up by the SR via the SR Ca^2+^ pump or effluxed out of the cell through the plasma membrane Na^+^/ Ca^2+^ exchanger, resulting in muscle relaxation [27]. As the amount of Ca^2+^ excluded by Na^+^/ Ca^2+^ exchanger is small in rodent myocytes, the SR Ca^2+^ pump activity significantly contributes to beat-to-beat Ca^2+^ depletion [27]. In the present study, 1% CSE treatment increased spike-shaped Ca^2+^ transients that were not triggered by electrical stimulation (secondary peaks) in some cardiomyocytes (S2 Fig), suggesting that spontaneous Ca^2+^ leakage from the SR via ryanodine receptors may have occurred. Furthermore, kinetic analysis during the decay phase of the Ca^2+^ transient showed that CSE rather promoted Ca^2+^ uptake into the SR (Fig 5), indicating that SR Ca^2+^ pump activity may have increased. This hypothesis was confirmed by preliminary experimental data showing that the phosphorylation level of Thr17 (which undergoes phosphorylation by CaMKII but not by PKA) of phospholamban, a regulator of the SR Ca^2+^ pump, was increased in the 1% CSE-treated group (S5 Fig). Indeed, alterations in the SR Ca^2+^ pump and/or its regulator, phospholamban have been reported in cardiomyocytes from mice exposed to cigarette smoke [28]. These results suggest that CSE increases SR Ca^2+^ pump activity via phospholamban phosphorylation and maintains SR Ca^2+^ content, thereby increasing systolic Ca^2+^ levels and Ca^2+^ transient amplitude. Diastolic Ca^2+^ levels are the result of a balance between Ca^2+^ release from the SR and Ca^2+^ uptake into the SR. Thus, intracellular Ca^2+^ dynamics were altered by CSE.

Although Ca^2+^ transient amplitude was increased by CSE treatment (Fig 4), fractional shortening was decreased (Fig 3). This discrepancy may be attributed to CSE dysregulating mitochondrial function, which is known to be relevant to cardiomyocyte contractility. Indeed, Fig 6 shows that mitochondrial membrane potential (ΔΨm), whose decrease is known as an early indicator of cell damage, is slightly but significantly reduced in CSE-treated cardiomyocytes compared to the Tyrode-treated group. To confirm whether mitochondrial dysfunction is indeed induced by CSE, other parameters involved in mitochondria-mediated cell death signaling were examined using cultured NRVMs showing contractile dysfunction and eventual cell death. Abnormal cytosolic Ca^2+^ overload induces mitochondrial Ca^2+^ influx via mitochondrial Ca^2+^ uniporters, both of which can lead to enhanced mitochondrial ROS production [29]. This can activate a large conductance channel in the mitochondrial inner membrane (IMM) known as mitochondrial permeation transition pore (mPTP), and opening of mPTP decrease ΔΨm [24], that collapse protonmotive force and inhibits ATP synthesis. Prolonged opening of the mPTP allows cytoplasmic solutes to flow into the mitochondria, causing massive swelling of the IMM, rupture of the outer membrane, that cause cytochrome c release and cell death [30]. We observed that mitochondrial ROS production was indeed increased in CSE treated NRVM (Fig 7). The opening of mPTP, suggested by reduced calcein fluorescence in mitochondria, was detected in CSE-treated cardiomyocytes compared to the Tyrode group (Fig 8). As observed in freshly isolated cardiomyocytes, ΔΨm detected by TMRE was also reduced by CSE treatment. Furthermore, immunofluorescence analysis showed that cytochrome c was released from mitochondria in the CSE treated group (Fig 9). These results strongly suggest that CSE causes mitochondrial dysfunction, leading to contractile dysfunction and cardiomyocyte death as observed in Figs 1–3. Similar CSE-induced mitochondrial abnormalities have been reported [31], and emerging evidence has demonstrated that mitochondrial dysfunction contributes to the contractile dysfunction of cardiomyocytes [32].

We observed that CSE concentrations of 0.1% or higher led to an eventual cessation of cardiomyocytes spontaneous beating (Fig 2). It is possible that various ion channel functions may be affected by CSE. For example, nicotine has been reported to reduce various myocardial K^+^ channel activities, including the transient outward current (I_to_/Kv4.3), delayed rectifier K^+^ current (I_Kr_/HERG) and inward rectifier K^+^ currents (I_K1_/Kir2.1) [33]. However, in our experiments using an *in vitro* expression system (S6 Fig), 1% CSE had no effect on the activities of G-protein-coupled inwardly rectifying K^+^ channels (GIRK1/GIRK4: the cardiac type heterotetramer), which is known to have significant influence on cardiac auto-rhythmicity. Further experiments are needed to clarify the impact of CSE on different channel activities. Another possibility is that mitochondrial dysfunction, which would disable energy generation [32], may contribute to the cessation of spontaneous beating of cardiomyocytes. We have previously reported that treating cultured cardiomyocytes with toxic insults that can cause mitochondrial damage, such as oxidative stress, results in the cessation of spontaneous beating of cardiomyocytes [34] and reduction in ΔΨ_m_ [17]. Loss of ΔΨ_m_ and mitochondrial Ca^2+^ overload leads to the rupture of the outer membrane, releasing intermembrane components such as cytochrome c, which induce cell death [35]. Thus, CSE-induced cardiomyocyte dysfunction (reduced contractility and spontaneous beating rate) and eventual cell death may be partially promoted by mitochondrial dysfunction.

Human cardiomyocytes are known to differ from rodent cardiomyocytes in the expression of several ion channels and in cellular electrophysiology and, hence beating rate. Therefore, we also examined the effects of CSE on human cardiomyocytes. We found that 5% CSE treatment ultimately reduced the spontaneous beating rate and contractility of human iPS-derived cardiomyocytes (Fig 10), similar to the observations in cultured rat cardiomyocytes (Fig 2). We first examined the effects of 1% CSE, which had little effect on human iPS-derived cardiomyocytes, not only in the 30 min treatment but also in the 90 min treatment. However, contractility decreased after 360 min of treatment, and spontaneous beating stopped after 24 h of treatment. These results indicate that CSE is toxic to human iPS-derived cardiomyocytes in a dose- and time-dependent manner. One reason why human iPS-derived cardiomyocytes appeared to be less sensitive to CSE than cultured rat cardiomyocytes could be that CSE was applied to Tyrode’s solution in NRVMs, whereas culture medium (maintaining medium) was used for human iPS-derived cardiomyocytes (because Tyrode’s solution is somewhat cytotoxic for these cells). The maintaining medium may contain protective factors (e.g., amino acids, growth factors, and proteins) that scavenge or counteract the various toxic compounds in the CSE. In human iPS-derived cardiomyocytes, 5% CSE treatment initially increased and then decreased the spontaneous beating rate of cardiomyocytes, whereas in cultured rat cardiomyocytes, only a decline was observed with 1% CSE treatment. The increase in spontaneous beating rate observed in human iPS-derived cardiomyocytes might be due to the effects of CSE on various K^+^ channels (which are differentially expressed in rat and human cardiomyocytes) [33].

Cigarette smoke contains thousands of chemicals, including nicotine, tar, carbon monoxide, and reactive oxygen species, some of which are highly reactive but unstable, while others are stable. Since the organs are exposed to a mixture of cigarette smoke, we considered it important to first examine the effects of the mixture itself. However, the question of which components of CSE are toxic is an important issue. Although we measured the nicotine concentration in the CSE as an indicator, previous studies have shown that cigarette smoke consists of a particle phase and a gas phase, and both tar and nicotine are present in the particle phase [36]. Gas-phase CSE (without nicotine or tar) is more likely to reach the circulation system and contains a variety of chemicals, including carbon monoxide, acetone, acrolein, and formaldehyde. A previous study using both LC/MS and GC/MS confirmed the presence of cytotoxic activity in CSE from Hi-Lite in two high-performance liquid chromatography fractions, from which five compounds—acrolein, methyl vinyl ketone, 2-cyclopenten-1-one, acetone, and propionaldehyde—were detected. In particular, acrolein and methyl vinyl ketone were found to be the major stable cytotoxic factors inducing acute cell membrane damage and cell death in C6 glioma cells [19]. Authentic standards of these compounds did indeed show cytotoxic activity, with a 50% effective rate (EC_50_) being 1/50th of the concentration of these compounds in CSE [19]. This suggests that some compounds other than nicotine in cigarette smoke, such as methyl vinyl ketone and acrolein, which are stable and can accumulate in the blood, can cause cytotoxicity *in vivo* even at fairly low concentrations. Whether these substances are indeed toxic to cardiomyocytes remains to be determined. In any case, the results of the present study are expected to provide important insights into the potential mechanisms of cytotoxicity associated with smoking.

## Conclusion

We determined that administration of CSE causes direct toxicity to cardiomyocytes. CSE induced abnormal intracellular Ca^2+^ dynamics and led to mitochondrial dysfunction, which may have caused contractile dysfunction and eventual cell death. In the future, it is necessary to elucidate more specific molecular mechanisms of the cardiotoxicity of CSE, which may lead to various drug discovery applications.

## Supporting information

S1 Fig Acute effects of CSE on the intracellular Ca^2+^ dynamics in freshly isolated adult rat ventricular myocytes. Intracellular Ca^2+^ transients of cardiomyocytes elicited by electrical field stimulation (1 Hz) at 37 °C before and after 6 min of 1% CSE treatment. (A) Representative traces for each group. (B) Summary of diastolic and systolic Ca^2+^ levels, and Ca^2+^ transient amplitude obtained from each trace are shown as mean ± S.E.M. (Vehicle: n = 25, 1% CSE: n = 31). **P<0.01 vs. 0 min of 1% CSE. ^#^*P*<0.05 vs. 6 min of Tyrode. ^##^*P*<0.01 vs. 6 min of Tyrode.
(PDF)

S2 Fig Abnormal Ca^2+^ dynamics detected in some ARVMs treated with CSE. (A) Traces of spike-shaped Ca^2+^ transients (arrowhead) were observed in cardiomyocytes treated with 1% CSE for 6 min. (B) The number of spike-shaped Ca^2+^ transients (during the 6 s measurement) are shown as mean ± S.E.M. (Vehicle: n = 42 cells, 1% CSE: n = 44 cells, from 4 experiments). N.D.: not detected. *P<0.05 vs. 6 min of Tyrode, ^##^*P*<0.01 vs. 0 min of 1% CSE.
(PDF)

S3 Fig Effect of 1% CSE on the spontaneous beating rate and contractility of human iPS-derived cardiomyocytes. Human iPS-derived cardiomyocytes incubated in culture maintaining medium were exposed to 1% CSE or corresponding amounts of PBS (37 °C), and contractile functions were measured at 0, 10, 20, 30, 60, 90 and 360 min. In another group of cells, the effects of 1% CSE were measured at 0, 24, and 48 h. (A) Time course of spontaneous beating rate (BPM: beats per minute) for each group. There was little difference between the two group. (B) Contraction deformation distance (CDD), an index of contractile force, was compared; 1% CSE was found to significantly decrease CDD after 360 min. C and D: The long-term (up to 48 h) effects of 1% CSE on (C) the spontaneous beating rate and (D) CDD of human iPS-derived cardiomyocytes. Human iPS-derived cardiomyocytes stopped spontaneous beating after 24 h. Data are shown as mean ± S.E.M. (PBS: n = 8, 1% CSE: n = 8). *P<0.05 vs. PBS-treated group, ^#^*P*<0.05 vs. 0 min of 1% CSE-treated group.
(PDF)

S4 Fig Effect of 0.1% CSE on the spontaneous beating rate and contractility of human iPS-derived cardiomyocytes. Human iPS-derived cardiomyocytes incubated in cultured maintaining medium were exposed to 0.1% CSE or corresponding amounts of PBS (37 °C), and contractile functions were measured at 0, 10, 20, 30, 60, and 90 min. In another group of cells, the effects of 0.1% CSE were measured at 0, 24, and 48 h. (A–D) Time course of spontaneous beating rate (BPM) and CDD of human iPS-derived cardiomyocytes were measured (A and B) up to 90 min and (C and D) up to 48 h. 0.1% CSE had little effect on each parameter.
(PDF)

S5 Fig Effect of 1% CSE on phosphorylation of phospholamban in freshly isolated adult rat ventricular myocytes. After 1% CSE or Tyrode treatment (20 min), total cell lysates of ARVMs were collected. Expression levels of total and phosphorylated phospholamban were detected by Western blotting. (A) Representative blots of phospho-phospholamban Ser16 (p-PLB Ser16), phospho-phospholamban Thr17 (p-PLB Thr17), and total-phospholamban (total-PLB) are shown. (B and C) Phosphorylation of (B) PLB Ser16 and (C) Thr17 was corrected for total PLB, and normalized phosphorylation relative to Tyrode is shown as mean ± S.E.M. (Vehicle: n = 3, 1% CSE: n = 3). Statistical analyses were performed using a paired Student’s t-test *P < 0.05 vs. Tyrode.
(PDF)

S6 Fig Effect of 1% CSE on GIRK currents exogenously expressed in *Xenopus* oocytes. Representative current traces in oocytes expressing GIRK1 and GIRK4 (A) before and (B) after application of 1% CSE. Currents were recorded in extracellular high K^+^ solution by the voltage protocol shown above the trace and repeated every 5 s. (C) Representative time course of current change at –100 mV (grey filled circle) and +40 mV (white filled circle) before and after 1% CSE application in extracellular solution. (D) Normalized current at –100 mV before (–) and after (+) application of 1% CSE from three cells. The current amplitude before CSE application was normalized to 1.
(PDF)

## References

[1] Forey BA, Thornton AJ, Lee PN. Systematic review with meta-analysis of the epidemiological evidence relating smoking to COPD, chronic bronchitis and emphysema. BMC Pulm Med. 2011;11:36. Epub 20110614. doi: 10.1186/1471-2466-11-36 . 21672193 PMC3128042

[2] Ambrose JA, Barua RS. The pathophysiology of cigarette smoking and cardiovascular disease: an update. J Am Coll Cardiol. 2004;43(10):1731–7. doi: 10.1016/j.jacc.2003.12.047 . 15145091

[3] Dikalov S, Itani H, Richmond B, Vergeade A, Rahman SMJ, Boutaud O, et al. Tobacco smoking induces cardiovascular mitochondrial oxidative stress, promotes endothelial dysfunction, and enhances hypertension. Am J Physiol Heart Circ Physiol. 2019;316(3):H639–h46. Epub 20190104. doi: 10.1152/ajpheart.00595.2018 . 30608177 PMC6459311

[4] Virdis A, Giannarelli C, Neves MF, Taddei S, Ghiadoni L. Cigarette smoking and hypertension. Curr Pharm Des. 2010;16(23):2518–25. doi: 10.2174/138161210792062920 . 20550499

[5] Aune D, Schlesinger S, Norat T, Riboli E. Tobacco smoking and the risk of sudden cardiac death: a systematic review and meta-analysis of prospective studies. Eur J Epidemiol. 2018;33(6):509–21. Epub 20180207. doi: 10.1007/s10654-017-0351-y . 29417317 PMC5995997

[6] Baba S, Iso H, Mannami T, Sasaki S, Okada K, Konishi M. Cigarette smoking and risk of coronary heart disease incidence among middle-aged Japanese men and women: the JPHC Study Cohort I. Eur J Cardiovasc Prev Rehabil. 2006;13(2):207–13. doi: 10.1097/01.hjr.0000194417.16638.3d . 16575274

[7] Messner B, Bernhard D. Smoking and cardiovascular disease: mechanisms of endothelial dysfunction and early atherogenesis. Arterioscler Thromb Vasc Biol. 2014;34(3):509–15. doi: 10.1161/ATVBAHA.113.300156 . 24554606

[8] Rival J, Riddle JM, Stein PD. Effects of chronic smoking on platelet function. Thromb Res. 1987;45(1):75–85. doi: 10.1016/0049-3848(87)90258-1 . 2951895

[9] Matetzky S, Tani S, Kangavari S, Dimayuga P, Yano J, Xu H, et al. Smoking increases tissue factor expression in atherosclerotic plaques: implications for plaque thrombogenicity. Circulation. 2000;102(6):602–4. doi: 10.1161/01.cir.102.6.602 . 10931797

[10] Csordas A, Bernhard D. The biology behind the atherothrombotic effects of cigarette smoke. Nat Rev Cardiol. 2013;10(4):219–30. Epub 20130205. doi: 10.1038/nrcardio.2013.8 . 23380975

[11] Eisner DA, Caldwell JL, Kistamás K, Trafford AW. Calcium and Excitation-Contraction Coupling in the Heart. Circulation research. 2017;121(2):181–95. doi: 10.1161/CIRCRESAHA.117.310230 . 28684623 PMC5497788

[12] Murakami D, Kono M, Nanushaj D, Kaneko F, Zangari T, Muragaki Y, et al. Exposure to Cigarette Smoke Enhances Pneumococcal Transmission Among Littermates in an Infant Mouse Model. Front Cell Infect Microbiol. 2021;11:651495. Epub 20210331. doi: 10.3389/fcimb.2021.651495 . 33869082 PMC8045753

[13] Mizutani N, Fuchikami J, Takahashi M, Nabe T, Yoshino S, Kohno S. Pulmonary emphysema induced by cigarette smoke solution and lipopolysaccharide in guinea pigs. Biol Pharm Bull. 2009;32(9):1559–64. doi: 10.1248/bpb.32.1559 . 19721232

[14] Ueha R, Ueha S, Kondo K, Nishijima H, Yamasoba T. Effects of Cigarette Smoke on the Nasal Respiratory and Olfactory Mucosa in Allergic Rhinitis Mice. Front Neurosci. 2020;14:126. Epub 20200218. doi: 10.3389/fnins.2020.00126 . 32132898 PMC7040099

[15] International Organization for Standardization. ISO 18145: Environmental tobacco smoke -Determination of vapour phase nicotine and 3-ethenylpyridine in air—Gas-chromatographic method 2003 [cited August 13, 2023]. https://www.iso.org/standard/33327.html.

[16] Nakamura TY, Jeromin A, Mikoshiba K, Wakabayashi S. Neuronal calcium sensor-1 promotes immature heart function and hypertrophy by enhancing Ca^2+^ signals. Circulation research. 2011;109(5):512–23. Epub 2011/07/09. doi: 10.1161/CIRCRESAHA.111.248864 . 21737792

[17] Nakamura TY, Nakao S, Wakabayashi S. Neuronal Ca^2+^ sensor-1 contributes to stress tolerance in cardiomyocytes via activation of mitochondrial detoxification pathways. J Mol Cell Cardiol. 2016;99:23–34. doi: 10.1016/j.yjmcc.2016.08.013 . 27555477

[18] Nakamura TY, Iwata Y, Arai Y, Komamura K, Wakabayashi S. Activation of Na^+^/H^+^ exchanger 1 is sufficient to generate Ca^2+^ signals that induce cardiac hypertrophy and heart failure. Circulation research. 2008;103(8):891–9. Epub 2008/09/09. doi: 10.1161/CIRCRESAHA.108.175141 . 18776042

[19] Noya Y, Seki K-i, Asano H, Mai Y, Horinouchi T, Higashi T, et al. Identification of stable cytotoxic factors in the gas phase extract of cigarette smoke and pharmacological characterization of their cytotoxicity. Toxicology. 2013;314(1):1–10. doi: 10.1016/j.tox.2013.08.015 23981515

[20] Nakao S, Wakabayashi S, Nakamura TY. Stimulus-dependent regulation of nuclear Ca^2+^ signaling in cardiomyocytes: a role of neuronal calcium sensor-1. PLoS One. 2015;10(4):e0125050. Epub 2015/04/22. doi: 10.1371/journal.pone.0125050 . 25897502 PMC4405540

[21] Minami I, Yamada K, Otsuji TG, Yamamoto T, Shen Y, Otsuka S, et al. A small molecule that promotes cardiac differentiation of human pluripotent stem cells under defined, cytokine- and xeno-free conditions. Cell Rep. 2012;2(5):1448–60. Epub 20121025. doi: 10.1016/j.celrep.2012.09.015 . 23103164

[22] Iseoka H, Miyagawa S, Fukushima S, Saito A, Masuda S, Yajima S, et al. Pivotal Role of Non-cardiomyocytes in Electromechanical and Therapeutic Potential of Induced Pluripotent Stem Cell-Derived Engineered Cardiac Tissue. Tissue Eng Part A. 2018;24(3–4):287–300. Epub 20170613. doi: 10.1089/ten.TEA.2016.0535 . 28498040 PMC5792250

[23] Henningfield JE, Stapleton JM, Benowitz NL, Grayson RF, London ED. Higher levels of nicotine in arterial than in venous blood after cigarette smoking. Drug Alcohol Depend. 1993;33(1):23–9. doi: 10.1016/0376-8716(93)90030-t . 8370337

[24] Rottenberg H. The Reduction in the Mitochondrial Membrane Potential in Aging: The Role of the Mitochondrial Permeability Transition Pore. Int J Mol Sci. 2023;24(15). Epub 20230801. doi: 10.3390/ijms241512295 . 37569671 PMC10418870

[25] Gustafsson AB, Gottlieb RA. Heart mitochondria: gates of life and death. Cardiovasc Res. 2008;77(2):334–43. Epub 20070821. doi: 10.1093/cvr/cvm005 . 18006487

[26] Wilson D, Wakefield M, Owen N, Roberts L. Characteristics of heavy smokers. Prev Med. 1992;21(3):311–9. doi: 10.1016/0091-7435(92)90030-l . 1614993

[27] Bers DM. Cardiac excitation-contraction coupling. Nature. 2002;415(6868):198–205. Epub 2002/01/24. doi: 10.1038/415198a . 11805843

[28] Hu N, Han X, Lane EK, Gao F, Zhang Y, Ren J. Cardiac-specific overexpression of metallothionein rescues against cigarette smoking exposure-induced myocardial contractile and mitochondrial damage. PLoS One. 2013;8(2):e57151. Epub 20130219. doi: 10.1371/journal.pone.0057151 . 23431404 PMC3576371

[29] Brookes PS, Yoon Y, Robotham JL, Anders MW, Sheu SS. Calcium, ATP, and ROS: a mitochondrial love-hate triangle. Am J Physiol Cell Physiol. 2004;287(4):C817–33. doi: 10.1152/ajpcell.00139.2004 . 15355853

[30] Bauer TM, Murphy E. Role of Mitochondrial Calcium and the Permeability Transition Pore in Regulating Cell Death. Circulation research. 2020;126(2):280–93. Epub 20200116. doi: 10.1161/CIRCRESAHA.119.316306 . 31944918 PMC8317591

[31] Yamada S, Zhang XQ, Kadono T, Matsuoka N, Rollins D, Badger T, et al. Direct toxic effects of aqueous extract of cigarette smoke on cardiac myocytes at clinically relevant concentrations. Toxicol Appl Pharmacol. 2009;236(1):71–7. Epub 20090127. doi: 10.1016/j.taap.2009.01.008 . 19371621

[32] Liu M, Lv J, Pan Z, Wang D, Zhao L, Guo X. Mitochondrial dysfunction in heart failure and its therapeutic implications. Front Cardiovasc Med. 2022;9:945142. Epub 20220824. doi: 10.3389/fcvm.2022.945142 . 36093152 PMC9448986

[33] Wang H, Shi H, Wang Z. Nicotine depresses the functions of multiple cardiac potassium channels. Life Sci. 1999;65(12):Pl143–9. doi: 10.1016/s0024-3205(99)00370-7 . 10503950

[34] Nakamura TY, Goda K, Okamoto T, Kishi T, Nakamura T, Goshima K. Contractile and morphological impairment of cultured fetal mouse myocytes induced by oxygen radicals and oxidants. Correlation with intracellular Ca^2+^ concentration. Circulation research. 1993;73(4):758–70. Epub 1993/10/01. . 8396508 10.1161/01.res.73.4.758

[35] Crompton M. The mitochondrial permeability transition pore and its role in cell death. The Biochemical journal. 1999;341 (Pt 2):233–49. Epub 1999/07/07. . 10393078 PMC1220352

[36] Pryor WA, Stone K. Oxidants in cigarette smoke. Radicals, hydrogen peroxide, peroxynitrate, and peroxynitrite. Ann N Y Acad Sci. 1993;686:12–27; discussion -8. doi: 10.1111/j.1749-6632.1993.tb39148.x . 8512242

