# Supplementary figures and images for: Direct toxicity of cigarette smoke extract on cardiac function mediated by mitochondrial dysfunction in Sprague-Dawley rat ventricular myocytes and human induced pluripotent stem cell-derived cardiomyocytes
Source: PLoS One. 2024 Jan 2;19(1):e0295737. doi: 10.1371/journal.pone.0295737 (PMC10760691; doi:10.1371/journal.pone.0295737)

**S1 Fig.**

**A**

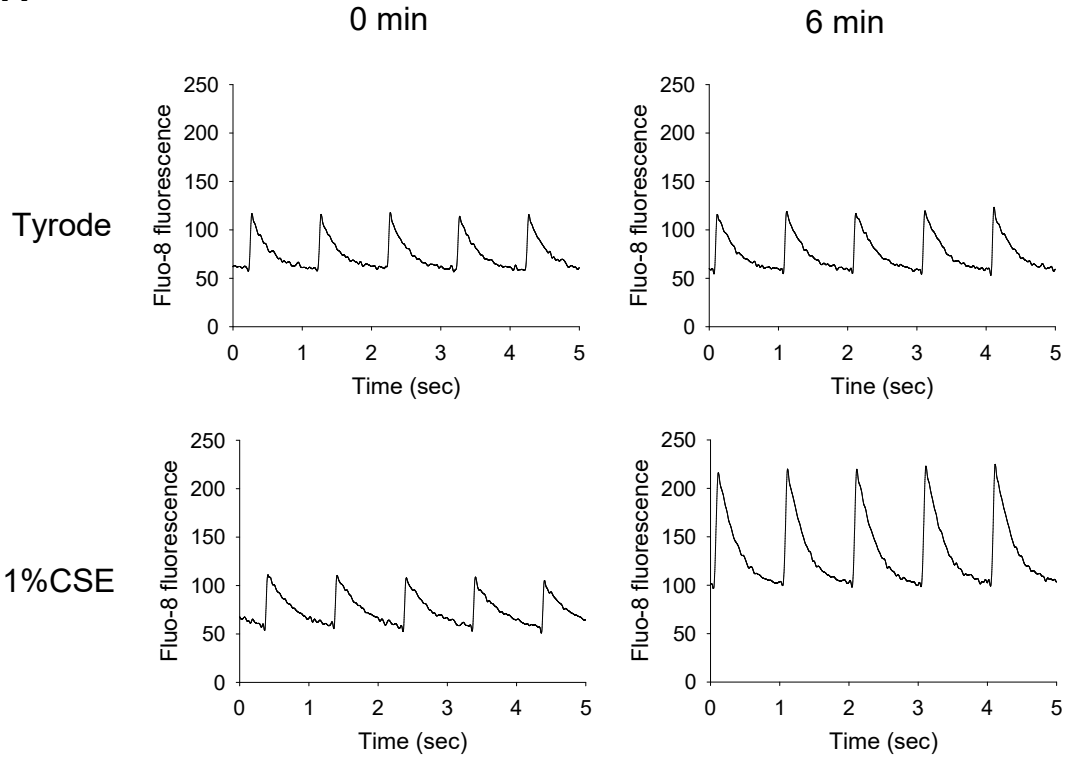

**B**

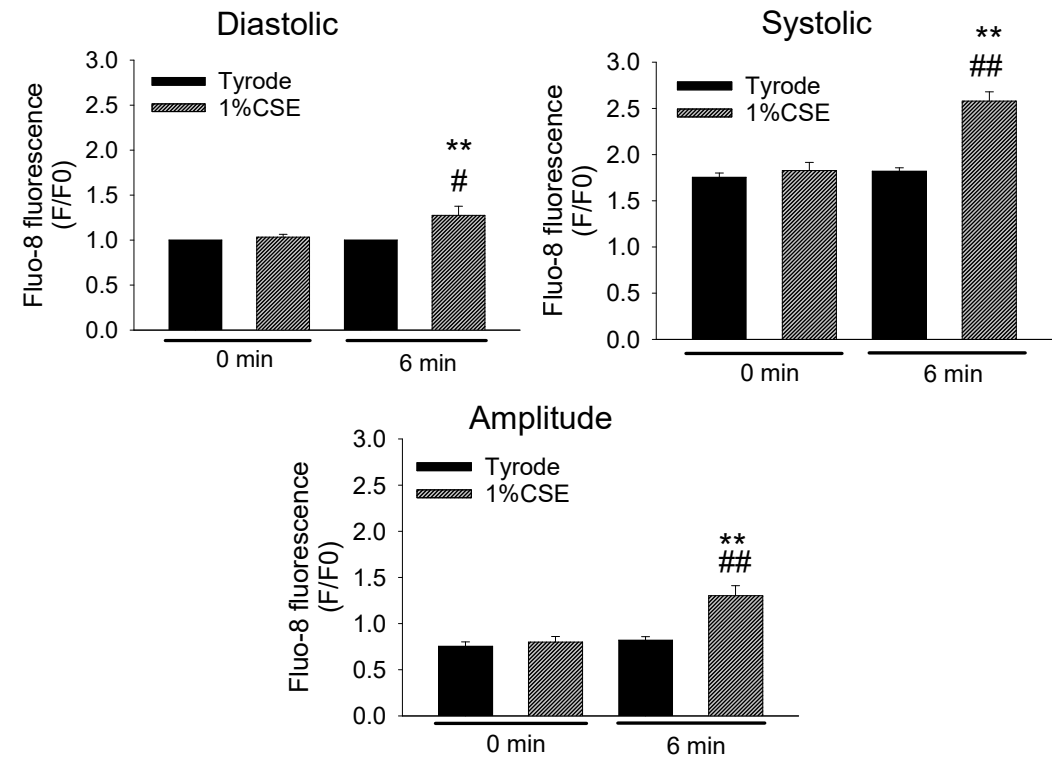

Supplement: S1 Fig — Intracellular Ca2+ transients of cardiomyocytes elicited by electrical field stimulation (1 Hz) at 37 °C before and after 6 min of 1% CSE treatment. (A) Representative traces for each group. (B) Summary of diastolic and systolic Ca2+ levels, and Ca2+ transient amplitude obtained from each trace are shown as mean ± S.E.M. (Vehicle: n = 25, 1% CSE: n = 31). **P<0.01 vs. 0 min of 1% CSE. #P<0.05 vs. 6 min of Tyrode. ##P<0.01 vs. 6 min of Tyrode. (PDF) [file pone.0295737.s001.pdf]

**S2 Fig.**

**A**

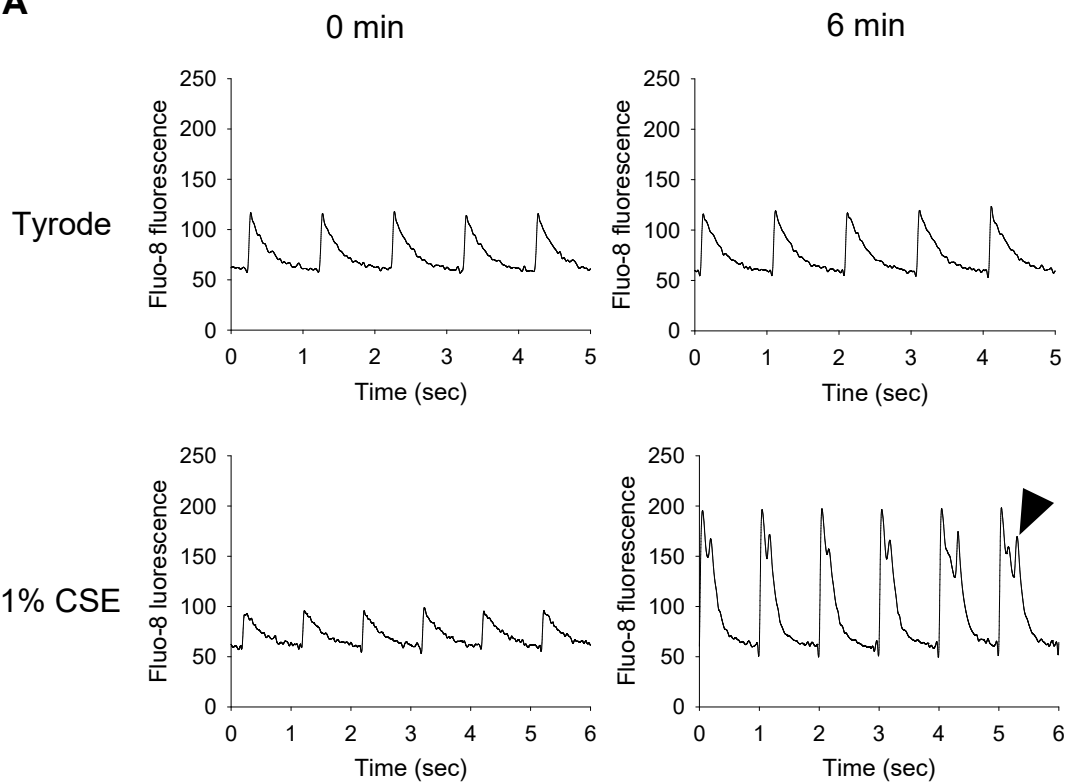

**B**

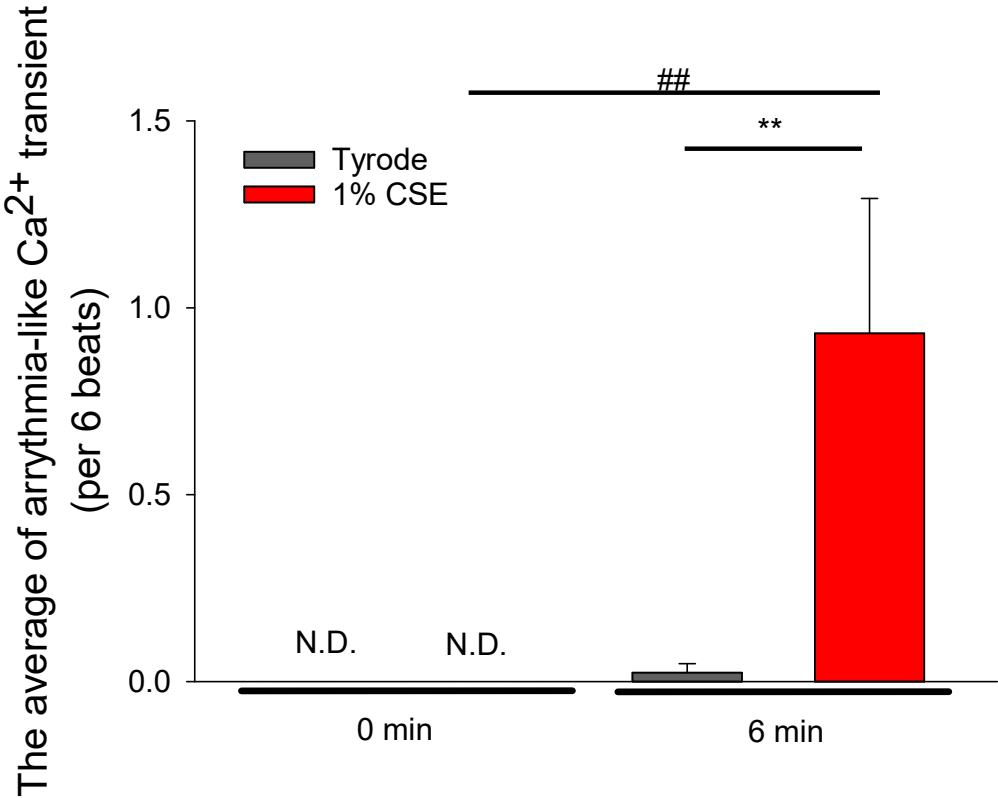

Supplement: S2 Fig — (A) Traces of spike-shaped Ca2+ transients (arrowhead) were observed in cardiomyocytes treated with 1% CSE for 6 min. (B) The number of spike-shaped Ca2+ transients (during the 6 s measurement) are shown as mean ± S.E.M. (Vehicle: n = 42 cells, 1% CSE: n = 44 cells, from 4 experiments). N.D.: not detected. *P<0.05 vs. 6 min of Tyrode, ##P<0.01 vs. 0 min of 1% CSE. (PDF) [file pone.0295737.s002.pdf]

**S3 Fig.**

**A**

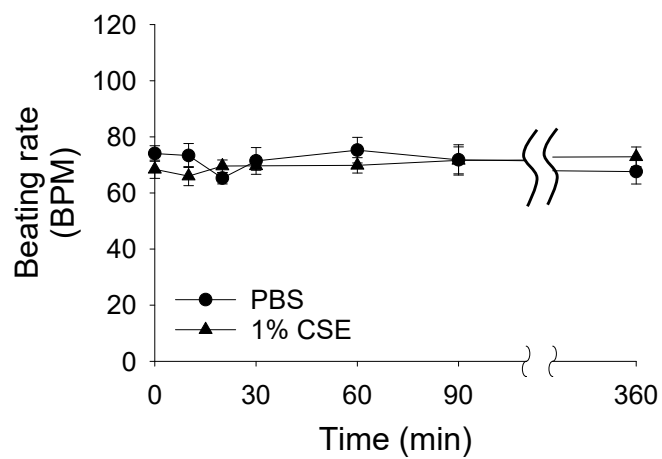

**B**

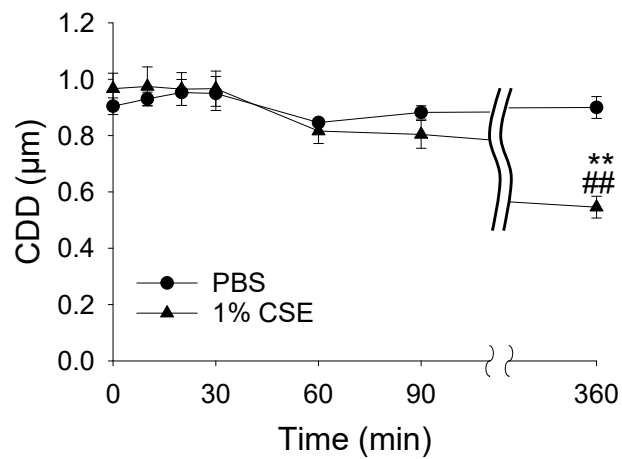

**C**

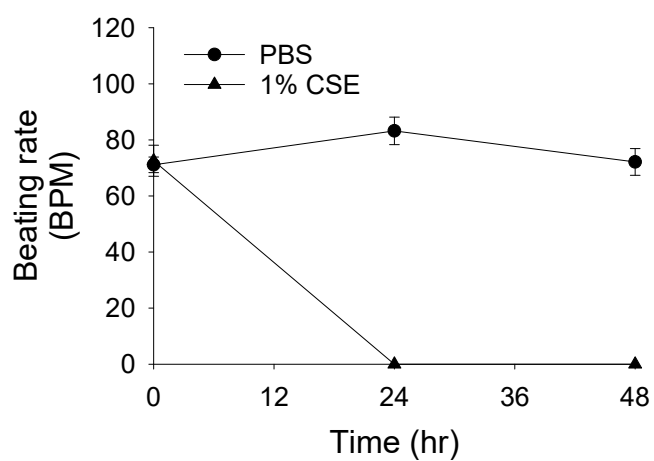

**D**

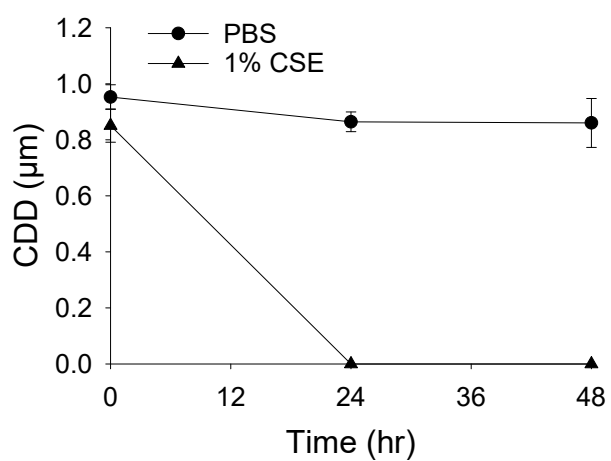

Supplement: S3 Fig — Human iPS-derived cardiomyocytes incubated in culture maintaining medium were exposed to 1% CSE or corresponding amounts of PBS (37 °C), and contractile functions were measured at 0, 10, 20, 30, 60, 90 and 360 min. In another group of cells, the effects of 1% CSE were measured at 0, 24, and 48 h. (A) Time course of spontaneous beating rate (BPM: beats per minute) for each group. There was little difference between the two group. (B) Contraction deformation distance (CDD), an index of contractile force, was compared; 1% CSE was found to significantly decrease CDD after 360 min. C and D: The long-term (up to 48 h) effects of 1% CSE on (C) the spontaneous beating rate and (D) CDD of human iPS-derived cardiomyocytes. Human iPS-derived cardiomyocytes stopped spontaneous beating after 24 h. Data are shown as mean ± S.E.M. (PBS: n = 8, 1% CSE: n = 8). *P<0.05 vs. PBS-treated group, #P<0.05 vs. 0 min of 1% CSE-treated group. (PDF) [file pone.0295737.s003.pdf]

**S4 Fig.**

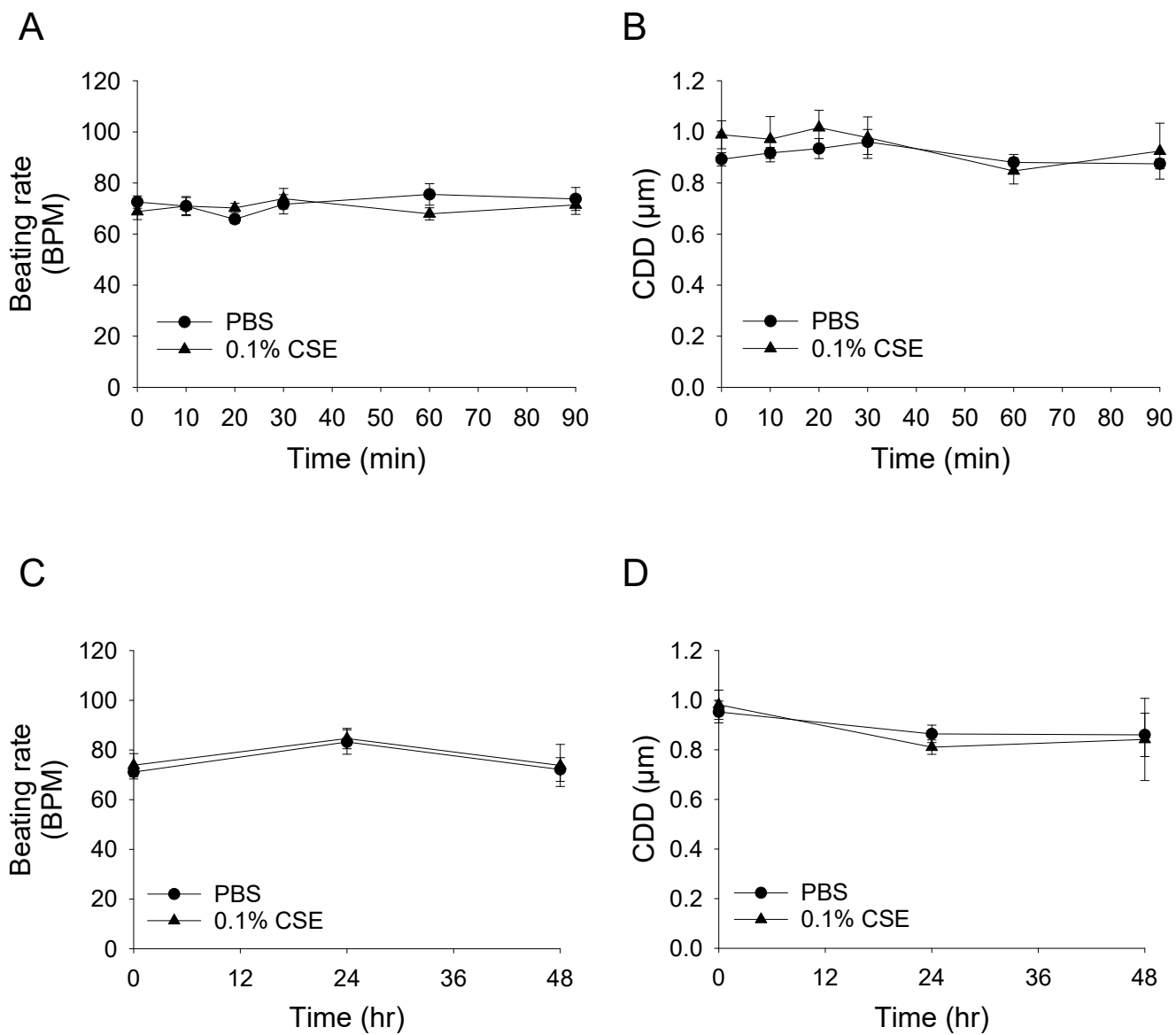

Supplement: S4 Fig — Human iPS-derived cardiomyocytes incubated in cultured maintaining medium were exposed to 0.1% CSE or corresponding amounts of PBS (37 °C), and contractile functions were measured at 0, 10, 20, 30, 60, and 90 min. In another group of cells, the effects of 0.1% CSE were measured at 0, 24, and 48 h. (A–D) Time course of spontaneous beating rate (BPM) and CDD of human iPS-derived cardiomyocytes were measured (A and B) up to 90 min and (C and D) up to 48 h. 0.1% CSE had little effect on each parameter. (PDF) [file pone.0295737.s004.pdf]

**S5 Fig.**

**A**

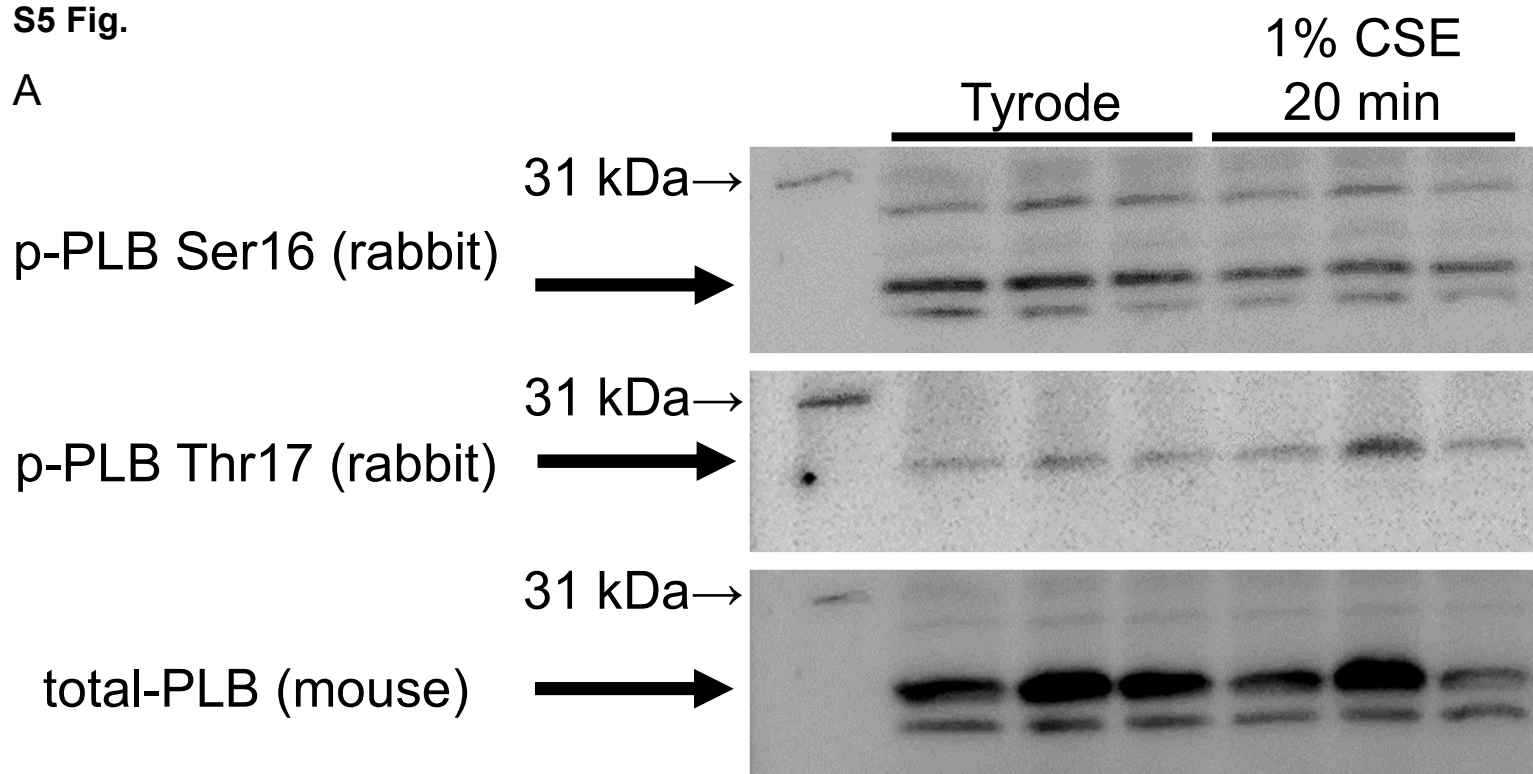

**B**

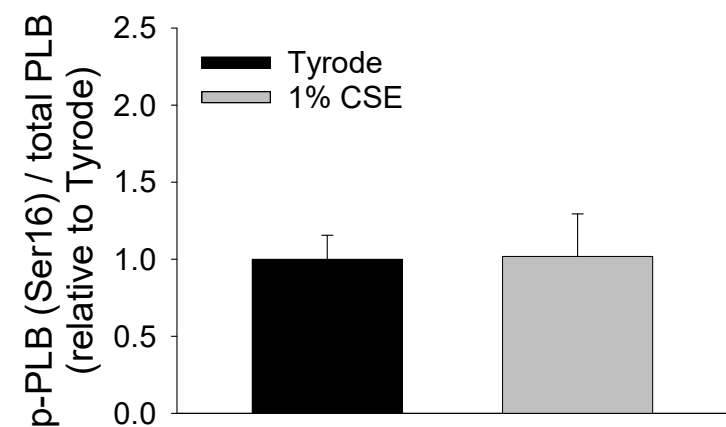

**C**

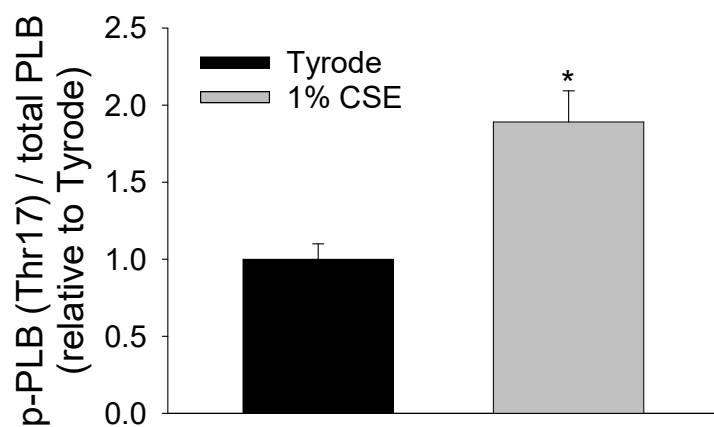

Supplement: S5 Fig — After 1% CSE or Tyrode treatment (20 min), total cell lysates of ARVMs were collected. Expression levels of total and phosphorylated phospholamban were detected by Western blotting. (A) Representative blots of phospho-phospholamban Ser16 (p-PLB Ser16), phospho-phospholamban Thr17 (p-PLB Thr17), and total-phospholamban (total-PLB) are shown. (B and C) Phosphorylation of (B) PLB Ser16 and (C) Thr17 was corrected for total PLB, and normalized phosphorylation relative to Tyrode is shown as mean ± S.E.M. (Vehicle: n = 3, 1% CSE: n = 3). Statistical analyses were performed using a paired Student’s t-test *P < 0.05 vs. Tyrode. (PDF) [file pone.0295737.s005.pdf]

**S6 Fig.**

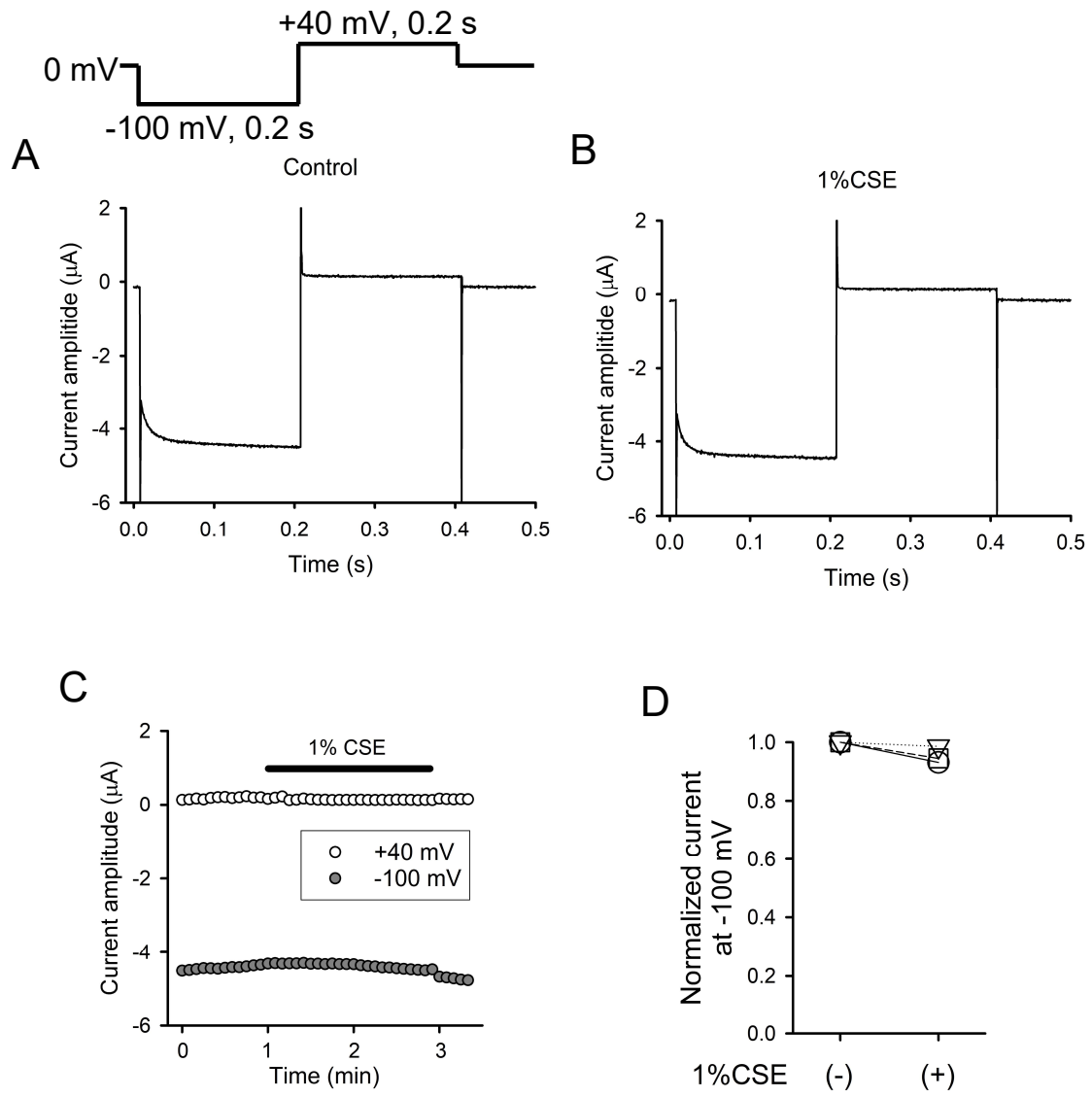

Supplement: S6 Fig — Representative current traces in oocytes expressing GIRK1 and GIRK4 (A) before and (B) after application of 1% CSE. Currents were recorded in extracellular high K+ solution by the voltage protocol shown above the trace and repeated every 5 s. (C) Representative time course of current change at –100 mV (grey filled circle) and +40 mV (white filled circle) before and after 1% CSE application in extracellular solution. (D) Normalized current at –100 mV before (–) and after (+) application of 1% CSE from three cells. The current amplitude before CSE application was normalized to 1. (PDF) [file pone.0295737.s006.pdf]
